# A half century of exploring DNA excision repair in chromatin

**DOI:** 10.1016/j.jbc.2023.105118

**Published:** 2023-07-30

**Authors:** Michael J. Smerdon, John J. Wyrick, Sarah Delaney

**Affiliations:** 1 Biochemistry and Biophysics, School of Molecular Biosciences, Washington State University, Pullman, Washington, USA; 2 Genetics and Cell Biology, School of Molecular Biosciences, Washington State University, Pullman, Washington, USA; 3 Department of Chemistry, Brown University, Providence, Rhode Island, USA

**Keywords:** DNA repair, chromatin, nucleosomes, cancer biology, histones, chromatin remodeling, aging

## Abstract

DNA in eukaryotic cells is packaged into the compact and dynamic structure of chromatin. This packaging is a double-edged sword for DNA repair and genomic stability. Chromatin restricts the access of repair proteins to DNA lesions embedded in nucleosomes and higher order chromatin structures. However, chromatin also serves as a signaling platform in which post-translational modifications of histones and other chromatin-bound proteins promote lesion recognition and repair. Similarly, chromatin modulates the formation of DNA damage, promoting or suppressing lesion formation depending on the chromatin context. Therefore, the modulation of DNA damage and its repair in chromatin is crucial to our understanding of the fate of potentially mutagenic and carcinogenic lesions in DNA. Here, we survey many of the landmark findings on DNA damage and repair in chromatin over the last 50 years (*i.e.*, since the beginning of this field), focusing on excision repair, the first repair mechanism studied in the chromatin landscape. For example, we highlight how the impact of chromatin on these processes explains the distinct patterns of somatic mutations observed in cancer genomes.

DNA damage can occur from endogenous species generated within cells during normal physiologic functions (*e.g*., respiration or inflammation) and from exogenous sources such as reactive chemicals or radiation in our environment. If this damage is allowed to persist, permanent mutations are introduced into the newly synthesized DNA of daughter cells. Importantly, these mutations can result in changes in gene function or expression that can lead to cancer and other diseases (1, 2, 3). However, cells are equipped with an extensive DNA-damage response (DDR) system to remove DNA damage and maintain genomic integrity. At the core of this system is an elaborate network of complementary DNA repair systems, each of which deals with specific classes of lesions (4, 5). These repair systems include direct damage reversal, excision repair, strand break repair, and interstrand crosslink repair. During the 1970s and 1980s, the majority of studies investigating DNA repair in chromatin focused on DNA excision repair or direct damage reversal by photolyase (6, 7, 8), and these studies followed closely after the discovery of nucleosomes (1973–1974) (9, 10). Therefore, we have limited the scope of this review to the area of excision repair in chromatin, realizing that significant work has also been done on direct damage reversal and, subsequently, on the repair of DNA strand breaks in chromatin. These latter studies will be covered extensively in another review that will appear elsewhere [Downs J, van Attikum H, Gasser SM (2023) Chromatin in Double-strand Break Repair, *in preparation*].

Damage of “naked DNA” (*i.e.*, DNA without bound proteins) has been studied in detail for many years and several excellent reviews have been published on this topic (11, 12). In the present review, we have focused on the influence of chromatin structure on both the distribution and yield of DNA damage and the efficiency of DNA repair in cells (6, 7, 8). In chromatin, the first level of packaging is a repeating array of nucleosomes, each consisting of a core particle (or NCP) containing ∼147 bp of DNA wrapped around an octamer of the core histones and linker DNA (∼40 bps, on average, in humans) (9, 13). In human diploid cells, ∼30 million nucleosomes are present, and these subunits restrict access to most of the genomic DNA. However, this packaging not only organizes DNA within nuclei but also facilitates the regulation of genomic processes such as transcription, replication, and repair. Indeed, changes to the epigenetic landscape of chromatin facilitate the recruitment of the protein machinery that mediates these processes (14).

Most damage in DNA is physically removed from the double helix and replaced with undamaged nucleotides. This pathway, called excision repair, occurs by either base excision repair (BER) or nucleotide excision repair (NER) (Fig. 1). Lesions removed by BER are typically small and non-helix-distorting base damage, including damage arising from depurination, cytosine deamination, alkylation, oxidation, etc. For example, BER is believed to be the main “housekeeping” pathway dealing with lesions that occur due to reactive oxygen species (ROS) generated during normal cell metabolism (4, 5). To repair such lesions, a variety of DNA glycosylases exist that recognize and excise specific classes of damaged bases. These glycosylases can be either monofunctional, with only glycosylase activity, or bifunctional, with glycosylase and ß-lyase activity (4, 15). In “short patch” BER, the abasic site remaining after glycosylase cleavage is a substrate for an AP endonuclease (APE1 in humans), which incises the DNA backbone generating a 3′ hydroxyl and leaves a deoxyribose phosphate (dRP) at the 5′-end (Fig. 1*A*, Short patch). This gap is processed by the 5′-dRP lyase and single nucleotide synthesis activities of DNA polymerase ß (Pol ß). The nick is then ligated by either DNA ligase one or a complex of DNA ligase three and X-ray repair cross-complementing protein 1 (XRCC1) (4, 15). In “long patch” BER, the gap generated by bifunctional glycosylases is cleaved by the 3′ phosphodiesterase of APE1 (Fig. 1*A*, Long patch). Then, Pol ß (in non-proliferating cells) or Pol *δ*/*ε* (in proliferating cells) synthesize two to ∼12 nts in a strand-displacement manner, followed by removal of the flap-by-flap endonuclease and ligation (16). Long patch-BER can also follow the activity of a monofunctional glycosylase if the abasic site is oxidized or alkylated preventing dRP lyase activity of Pol ß (17).

**Figure 1:**
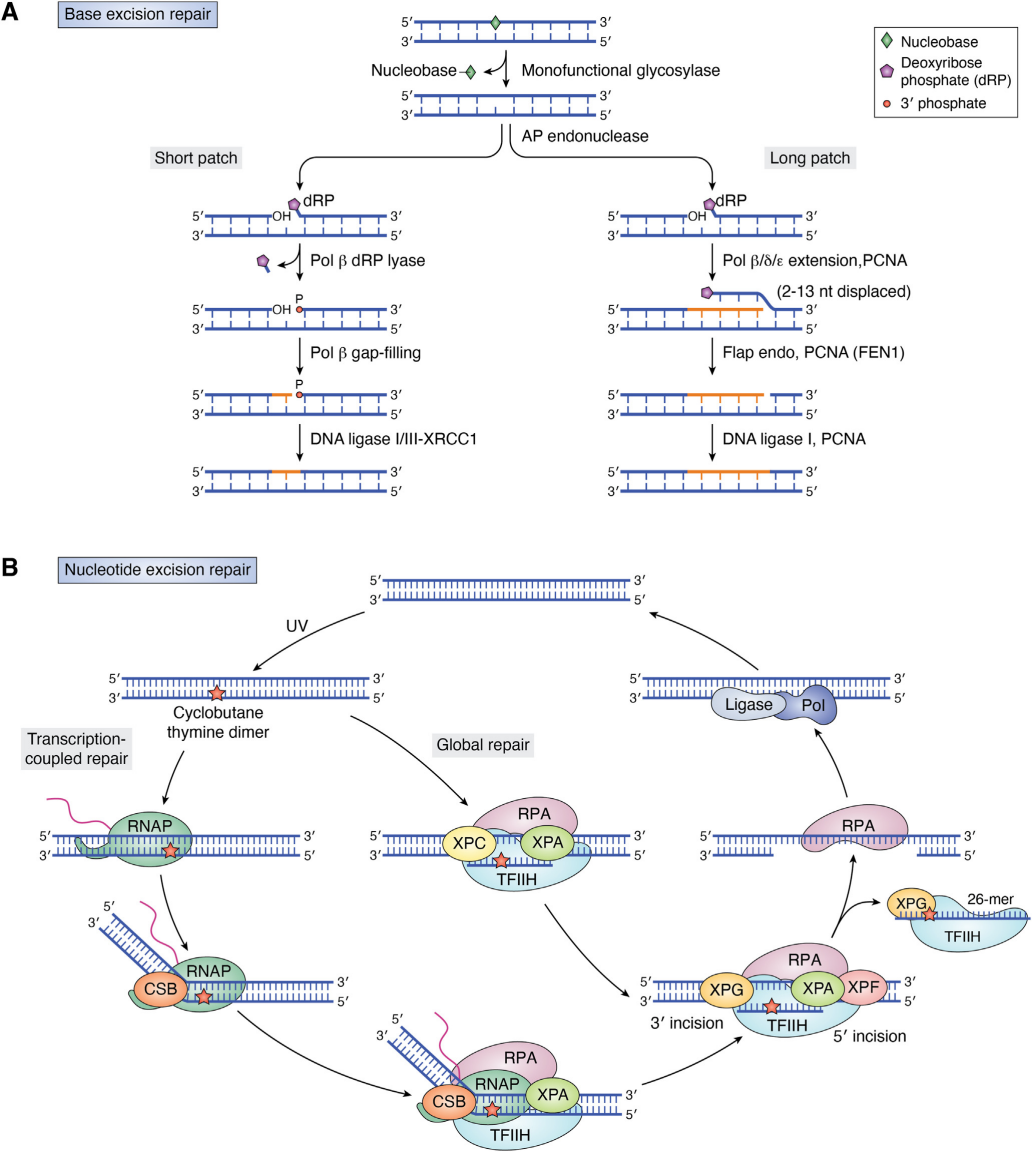
**Mechanisms of excision repair.***A*, base Excision Repair (BER) following mono-functional glycosylase activity. See text for details. [Modified figure from Dr Yesenia Rodriguez, National Institute of Environmental Health Sciences, who adapted it from (240)] *B*, nucleotide Excision Repair (NER) in humans [Modified from Figure 1*B* of (22)]. See text for details. CS, *Cockayne syndrome*; Pol, DNA polymerase; RNAP, RNA polymerase; RPA, replication protein A; TCR, transcription-coupled repair; TFIIH, transcription factor IIH; XP(A,C,G or F), *xeroderma pigmentosum* group A,C,G or F protein.

In contrast, nucleotide excision repair is responsible for repairing bulky DNA-distorting lesions caused primarily by exogenous sources including UV radiation (Fig. 1*B*). There are two major sub-pathways of NER: global genome NER (GG-NER) and transcription-coupled NER (TC-NER). In GG-NER, the main damage sensor in human cells is the XPC (Xeroderma Pigmentosum, complementation group C) protein, complexed with RAD23 B (UV excision repair protein Radiation sensitive 23B) protein and CETN2 (Centrin 2). This complex scans DNA for transient ssDNA regions caused by disrupted base pairing due to the lesion (18, 19). In the case of UV-induced cyclobutane pyrimidine dimers (CPDs), the UV–DDB (UV DNA damage-binding protein) complex, consisting of DDB1 (XPE-binding factor) and the GG-NER-specific protein DDB2, directly binds UV-induced lesions (20). The XPC-bound lesion becomes the substrate for the transcription initiation factor II H (TFIIH) complex, which functions in NER to unwind the DNA helix and verify that a lesion is present (Fig. 1*B*, Global repair) (3, 21, 22). It is worth noting that a recent report indicates that a minor amount of GG-NER activity persists even in the absence of XPC (23), although the mechanism responsible for XPC-independent GG-NER is unclear.

During the 1990's, the incision step of GG-NER was reconstituted *in vitro* with purified yeast proteins by the Prakash group at the University of Texas Medical Branch in Galveston (reviewed in (24)). In a landmark paper, Guzder *et al*. (25) established that Rad14, RPA, the Rad4–Rad23 complex, TFIIH, Rad2, and the Rad1-Rad10 complex mediates the formation of dual incisions at specific sites 5′ and 3′ from either a UV-induced photoproduct or an N-acetoxy-2-aminoacetylfluorene adduct to generate a single strand damage-containing DNA fragment 24 to 27 nts long, which almost certainly revealed the formation of a bubble structure containing the lesion prior to dual incision (24). In human cells, the incision step involves activities of structure-specific endonucleases (XPF–ERCC1 and XPG) to cut the damaged strand at specific sites 5′ and 3′ to the lesion, respectively, resulting in an excised single strand fragment of 25 to 28 nts (26, 27), mirroring this activity in yeast. Finally, the replication proteins PCNA (proliferating cell nuclear antigen), RFC (replication factor C), Pol *δ*, Pol *ε*, or Pol *κ*, and DNA ligase one or XRCC1– DNA ligase three carry out the final step of gap-filling synthesis and ligation. The choice of polymerase is determined by the state of proliferation of the cell.

The TC-NER pathway is initiated by RNA Pol II stalling at a bulky lesion on the transcribed strand (TS) (Fig. 1*B*, Transcription-coupled repair). During transcription elongation UV-stimulated scaffold protein A (UVSSA), ubiquitin-specific-processing protease 7 (USP7), and Cockayne syndrome protein (CSB) only transiently interact with RNA Pol II. However, the affinity of CSB for stalled RNA Pol II increases when RNA Pol II stalls at a DNA lesion (3, 28). CSB forms a complex with the Cockayne syndrome WD repeat protein CSA, which triggers the assembly of other TC-NER components (29), including the core NER proteins and TC-NER specific proteins, such as XAB2 (XPA-binding protein 2) and nonhistone protein HMGN1 (19). Furthermore, two different laboratories showed that elongation factor ELOF1 has an evolutionarily conserved role in TC-NER, where it promotes the recruitment of the TC-NER factors UVSSA and TFIIH to efficiently repair transcription-blocking lesions (30, 31). Additionally, ELOF1 modulates transcription to protect cells against transcription-mediated replication stress, thereby preserving genome stability (30, 31). Once localized at the lesion site, RNA Pol II may be backtracked or evicted to expose the damaged region of DNA. TFIIH is then recruited to the lesion, and the next series of events are thought to be identical to GG-NER removal of the lesion from the TS. Finally, it remains unresolved if, following TCR at damage sites, the majority of RNAPII complexes are displaced (22) or continue to elongate the truncated RNA (28).

In this review, we survey many landmark findings on DNA damage and excision repair in chromatin over the last half century (*i.e.*, since the beginning of this field). We regret that several important studies by our colleagues were not able to be discussed and/or cited due to the large scope of this topic and journal space limits.

## Modulation of the distribution and yield of DNA damage in chromatin

### DNA damage in nucleosomes

Early on it was clear that different classes of DNA lesions form either preferentially in nucleosome linker DNA and open regions of chromatin or about equally (per unit DNA) in linker and core regions (32). As expected, DNA lesions caused by bulky damaging agents (*e.g*., bleomycin-induced strand breaks, trimethylpsoralen (TMP) crosslinks, aflatoxin B1and benzo[*a*]pyrene-diol-epoxide (BPDE) adducts) show a marked preference for linker DNA (reviewed in (32)). Even certain small alkylating agents (*e.g*., methyl nitrosourea) show this structural bias (33), suggesting that agent size is not the only factor determining the preferred lesion sites in chromatin; nevertheless, *most* small alkylating agents do not show a bias. For example, dimethyl sulfate, which forms N^7^-methylguanine in the major groove and N^3^-methyladenine in the minor groove, produces a similar alkylation pattern in either isolated or reconstituted nucleosomes and their corresponding naked DNA (34, 35). These results indicate that the dynamic nature of nucleosomes allows DNA bases to be accessible to many small DNA alkylating agents in both the major and the minor grooves.

Bifunctional alkylating agents, like cisplatin, can form intra- and interstrand crosslinks in DNA (36). The alkylation patterns induced by these crosslinkers show similar preferences for modifying Guanines in nucleosomes (37). In addition, it was shown decades ago by electron microscopy (EM) that TMP photo-crosslinking of DNA in chromatin occurs in linker DNA and nucleosome-free regions (38, 39). Thus, virtually all the bifunctional alkylating agents that form interstrand crosslinks in DNA have been shown to have a substantial bias for crosslinking nucleosome linker and nucleosome-free regions in chromatin.

**Figure 2:**
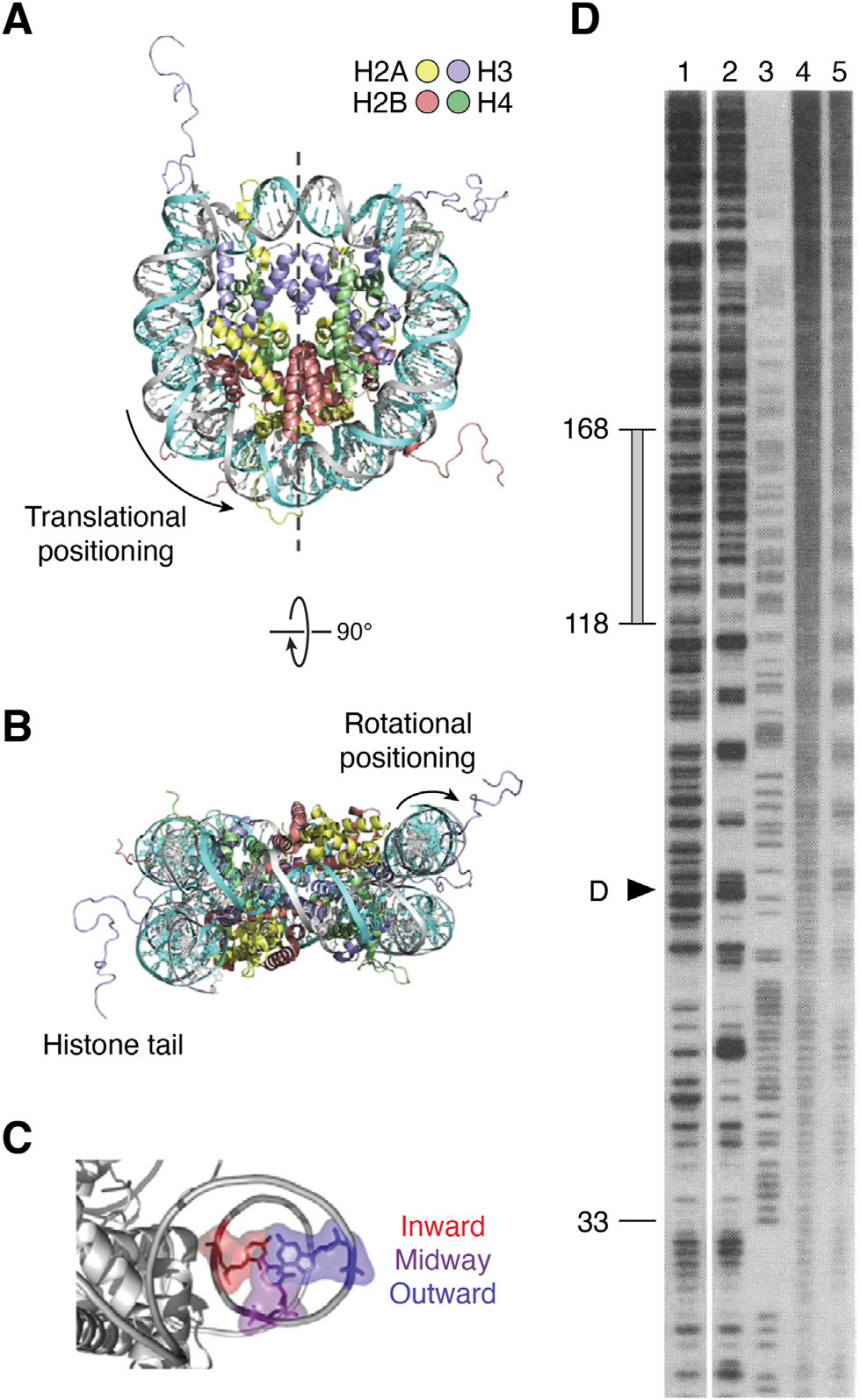
**Orientation of DNA in NCPs.***A*, top view of NCP looking down the dyad axis. Crystal structure of NCP (PDB 1kx5), looking down the superhelical axis of the 147 bp of DNA wrapped around a histone octamer: H2A (*yellow*), H2B (*red*), H3 (*blue*), and H4 (*green*). The dyad axis is denoted as a *dashed line*. Translational positions of DNA bases are described relative to their displacement from the central base in the NCP. *B*, side view of NCP looking down the dyad axis. Rotational positioning of DNA bases is described relative to the center of the histone core. *C*, rotational positions of three DNA bases. Outward-facing, midway-facing, and inward-facing positions are denoted in blue, purple, and red, respectively. Histones are colored gray, and just one DNA strand is shown for clarity. Modified from Figure 2 of (126). *D*, Hydroxyl radical footprints of NCP formed on *X. borealis* 5S rDNA. Cleavage patterns are for 5S rDNA, labeled on the noncoding strand, generated by DNase I (lane 1) and •OH (lane 4); and for rDNA NCPs generated by DNase I (lane 2) and •OH (lane 5). Lane three contains G + A markers from the chemical cleavage of 5S rDNA. DNA fragments were separated on a denaturing gel. Positions of the NCP dyad axis and internal control region of the 5S rRNA gene (*gray bar* covering positions +45 to +95) are shown on the left side of the gel (modified from Figure 1*B* of (41)).

Free radicals are a class of DNA-damaging agents that are continuously formed in cells (4, 5). These radicals are extremely reactive with DNA bases and create DNA strand breaks in chromatin. Hydroxyl radical (•OH) induced DNA strand breaks have proven to be a useful tool in chromatin research as they show only modest DNA sequence selectivity. This feature led to the popular “hydroxyl radical footprinting assay” developed to study the interactions between DNA and DNA-binding proteins (40). Indeed, cleavage of DNA in nucleosomes by •OHs displays an ∼10.5 base periodicity, reflecting the rotational setting of a DNA strand on the histone surface (Fig. 2, panels *A*–*C*). The rotational setting of the DNA strand is described as inward (**In**) for regions where the DNA minor groove faces the histones, outward (**Out**) where the minor groove strand faces solution, or midway (**Mid**) for positions in between (Fig. 2*C*). The more cleavable DNA locations in the hydroxyl radical footprint are those facing outward toward the solvent and away from the histones (41) (Fig. 2*D*, lane 5). Thus, histones play a major role in reducing the overall yield of strand breaks in chromatin relative to naked DNA.

**Figure 3:**
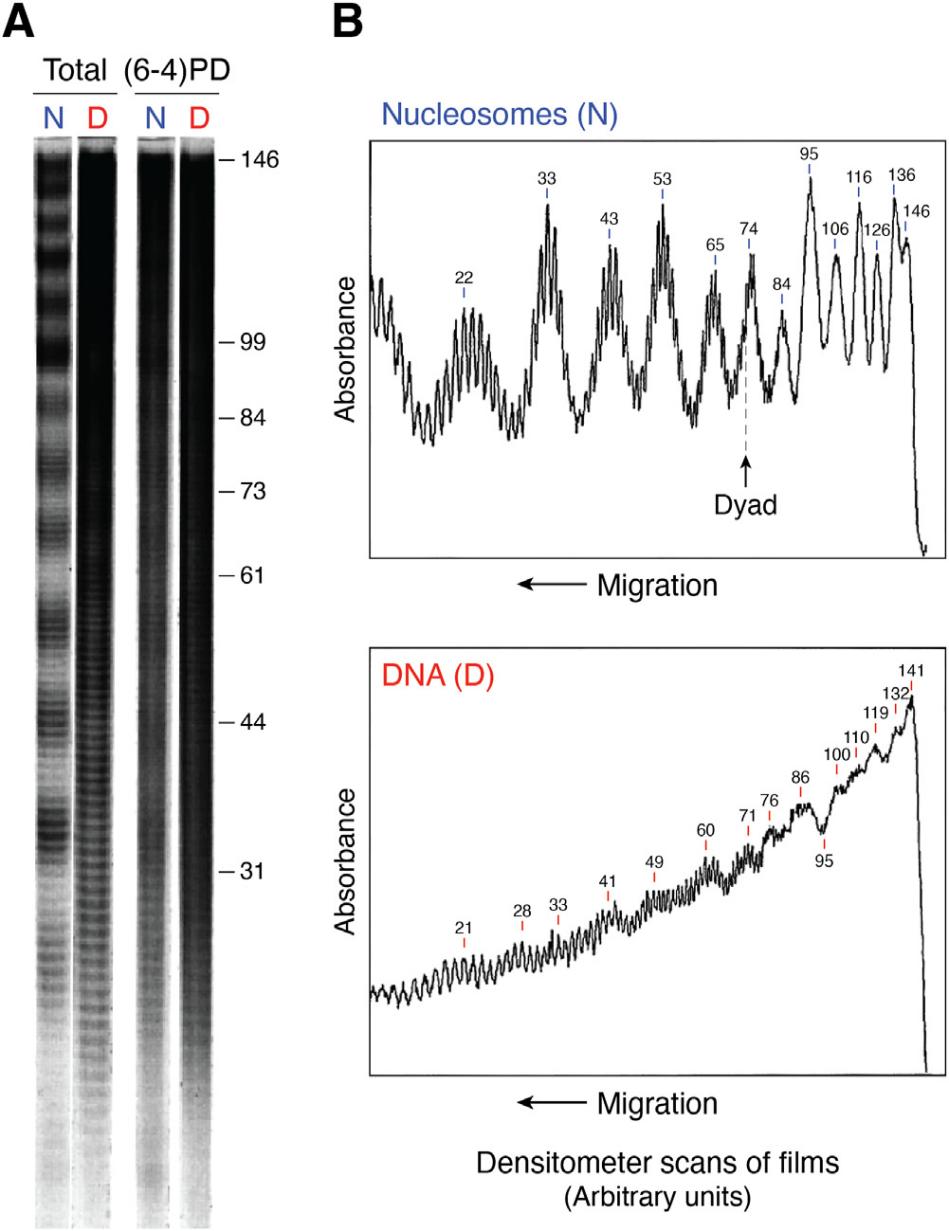
**UV photofootprint of nucleosome core DNA.***A*, denaturing gels of 5’end-labeled NCP DNA, digested with T4 DNA polymerase-exonuclease before and after photoreversal of CPDs with UV photolyase. CPD & (6–4)PP, no photoreversal; (6–4)PP Only, with photoreversal. N, nucleosome; D, DNA. *B*, scans of T4 DNA polymerase-exonuclease digestion profiles of UV-irradiated nucleosomes (*upper panel*) and DNA (*lower panel*). Numbers show distance (in bases) from the 5′ end of NCP DNA (*arrow denotes dyad*) (For details, see (47)).

The formation of UV photoproducts is also greatly influenced by the structure of DNA in chromatin (6, 42, 43). However, unlike bulky lesions, the major UV photoproduct (CPD) forms almost randomly between linker and core regions of nucleosomes (44, 45), while it was originally reported that pyrimidine (6–4) pyrimidone photoproducts [or (6–4)PPs] have a stronger bias for formation in linker DNA and nucleosome-free regions (46). Smerdon and colleagues at Washington State University used a T4 polymerase-exonuclease blockage assay to detect the distribution of these photoproducts *within* NCPs at nucleotide resolution (47). They showed there is a striking periodic pattern of CPD formation in NCPs from irradiated cells, irradiated chromatin, or NCPs irradiated *in vitro*, with an *average* periodicity of 10.3 ± 0.1 bases (Fig. 3). As with the •OH footprint (Fig. 2*D*), this “UV photofootprint” reflects the rotational setting of DNA on the histone surface, where peak levels of CPD formation occur where the DNA minor groove is facing out from the histone surface (47).

The UV photo footprint of NCPs appears to reflect the bending of DNA around histones, creating structural constraints on the DNA flexibility (*e.g*., roll and propeller twisting) [see discussion in (6)]. Indeed, Wyrick and coworkers at Washington State University recently analyzed ∼180 high-resolution nucleosome structures to characterize the role of both DNA flexibility and DNA conformation in CPD formation (48). Their results demonstrate that the sharp bending of DNA around histones (49) results in conformations more susceptible to CPD formation at positions where the minor groove faces out toward the solvent (48). This study provides strong evidence that the mechanism most responsible for the periodic modulation of UV-induced CPD formation in nucleosome DNA is the variable DNA conformation on the histone surface of NCPs.

Over the past decade, several approaches were developed to map UV-induced lesions across entire genomes of cells [reviewed in (50, 51)]. Initially, anti-CPD antibodies were used to immunoprecipitate lesion-containing DNA fragments, which were detected using tiling microarrays (52, 53). These studies showed how DNA sequence can influence UV-induced damage formation. Furthermore, a microarray-based method demonstrated that chromatin structure in yeast ensures efficient removal of DNA damage by GG-NER and that Abf1 binding sites provide locations where GG-NER is organized to promote efficient genomic DNA repair (54).

These microarray-based methods, however, fell short of mapping DNA lesions at single nucleotide resolution.

**Figure 4:**
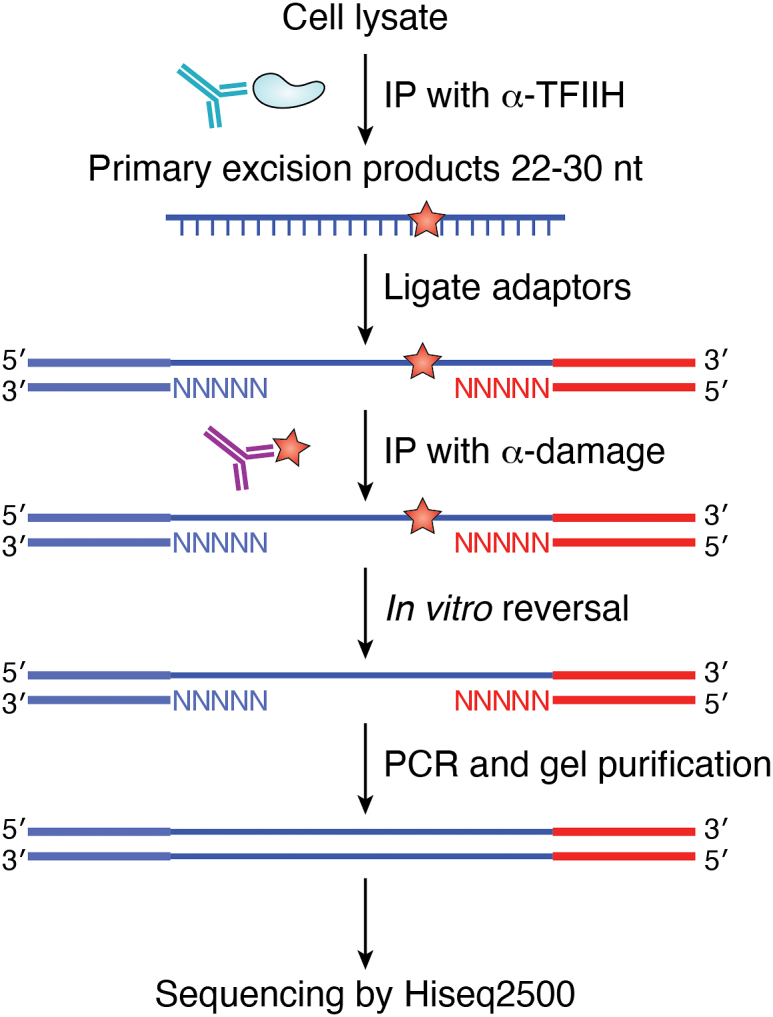
**Schematic of XR-seq method for high-resolution genome-wide mapping of DNA repair** [Modified from Figure 2*B* of (51)]. The key step is the capture of the excised oligomer by TFIIH co-immunoprecipitation followed by damage-specific immune-precipitation (IP). See text for details.

The advent of next-generation sequencing revolutionized the mapping of UV-induced damage at high resolution across the genome. Sancar and coworkers at the University of North Carolina developed “excision repair sequencing” (or XR-seq) (55), which utilizes TFIIH co-immunoprecipitation followed by damage-specific immunoprecipitation to capture the ∼25 to 30 nucleotide fragments excised during NER (Fig. 4). This method has proven to be a powerful method to map the repair of DNA lesions across the genome (55, 56). We note that for his contributions to our understanding of the mechanisms of NER and photoreactivation of UV photoproducts, Aziz Sancar was a co-recipient of the 2015 Nobel Prize in Chemistry, with Tomas Lindahl and Paul Modrich, for mechanistic studies on DNA repair (57).

The Wyrick group subsequently developed a high-resolution method, called CPD-seq (58), where UV irradiated DNA is sonicated into small fragments, ligated to a double-stranded DNA adapter, and treated with terminal transferase (and dideoxy-ATP) to yield DNA fragments where the free 3′-OH groups are eliminated. The DNA is then digested with T4 endo V and APE1 to generate new 3’-OH groups immediately upstream of the CPD lesion. These fragments are then ligated to a biotinylated second adaptor DNA, to allow purification of the ligated fragments. The CPD-seq library that is generated is amplified with primers complementary to the two adaptors and subjected to next-generation sequencing. Thus, one can map CPD formation across the genome at single nucleotide resolution. In addition, CPD maps generated at different repair times can be used to investigate the time course of CPD removal genome-wide (58).

The XR-seq and CPD-seq methods complement each other to form a valuable set of tools for mapping genome-wide repair of UV damage in DNA. Overlaying the CPD-seq data onto a well-defined map of yeast nucleosome positions (59) revealed that yeast nucleosomes *in vivo* induce a strong UV photofootprint. The peaks of CPD formation (after normalizing for dipyrimidine content) coincide with outward rotational settings in the NCP, exhibiting a striking periodicity of ∼10 bp (58) that closely mirrors the UV photofootprint previously observed in UV-irradiated mammalian cell chromatin (47) (Fig. 3). Notably, the CPD-seq generated UV photofootprint was most apparent within strongly positioned NCPs in yeast (∼10,000 nucleosomes), but was barely detectable among weakly positioned NCPs (∼7500 nucleosomes) (58). The lack of a uniform rotational setting among weakly positioned NCPs likely masks the UV photofootprint at these locations.

**Figure 5:**
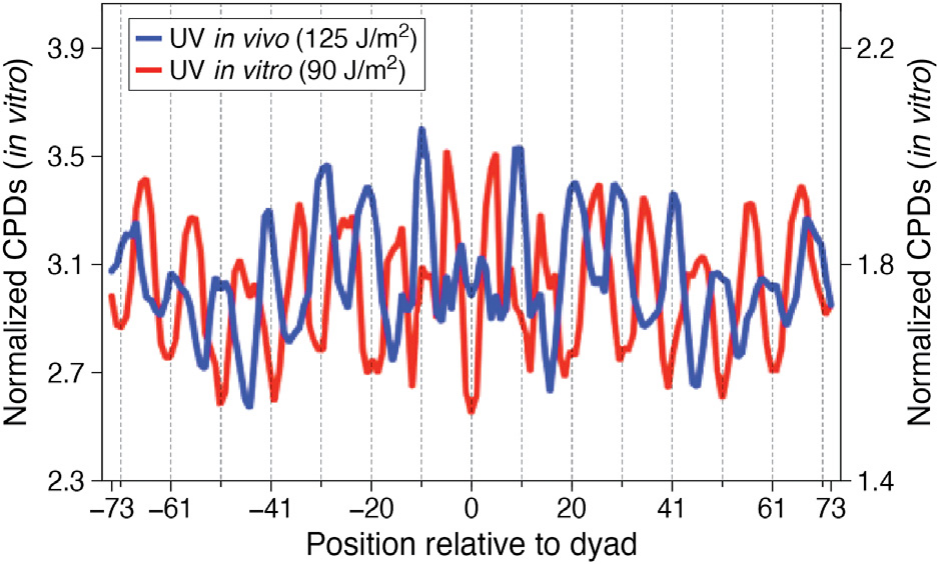
**Distribution of CPDs in UV-irradiated (naked) DNA and DNA within nucleosomes.** Yeast genomic DNA was either irradiated *in vitro* (*red line*) with 90 J/m^2^ UVC light or in intact cells (*blue line*) with 125 J/m^2^ UVC light. These doses were chosen because they yielded similar DNA damage levels in each case. *Red peaks* show that CPD formation occurs more frequently in DNA that adopts an inward rotational setting *in vitro* (*dotted vertical lines*), whereas CPD formation *in vivo* (0-h UV sample) shows the opposite trend. Modified from (58).

It should be noted that NCP DNA has a distinct sequence bias, where A-T-rich sequences tend to position at **In** rotational settings, while G-C-rich sequences tend to adopt **Out** rotational settings (60). Therefore, TT dinucleotides, which are the most prone to forming CPD lesions, tend to be positioned at **In** rotational settings [*e.g.*, (58, 59)]. This bias of TT’s in NCP DNA is clearly shown by the CPD levels in UV-irradiated *naked* NCP DNA (Fig. 5, red line). However, the opposite pattern occurs when this DNA is packaged into nucleosomes (Fig. 5, blue line). Thus, TT-rich DNA sequences at **In** rotational settings in NCPs are essentially “shielded” from UV damage, presumably reflecting the DNA conformational constraints discussed earlier. Mao and coworkers hypothesized that this mechanism operates in all eukaryotes and may be an important modifier of UV-induced mutagenesis (58).

The distribution of (6–4)PPs in UV-irradiated chromatin differs from that of CPDs, having a less striking periodicity *within* NCPs [Fig. 3; see also (46)]. These differences may, at least partially, reflect the difference in the photochemistry of the two lesions (61, 62). In addition, the overall levels of (6–4)PPs in UV-irradiated chromatin are significantly less than that of CPDs (61). However, the yield of (6–4)PPs can be much higher at *specific sites* in chromatin, such as the promoter region of the active *PGK1* gene (63), which increases their impact on UV-induced mutagenesis at specific sites in mammalian cells (64).

Unexpectedly, there is little change in the DNA structure around the damaged region in a CPD-containing NCP (65) while, as expected, the region surrounding a (6–4)PP-containing NCP is structurally disordered (66). Therefore, the more constrained NCP DNA is expected to be less capable of conforming to (6–4)PP structures, as compared to linker DNA in chromatin. Indeed, a nonuniform distribution of (6–4)PPs in chromatin was observed in early studies (44, 45), which provided a partial explanation for the more rapid repair of these lesions (see Alteration in chromatin structure during DNA excision repair). However, Wyrick’s lab has recently shown the distribution of (6–4)PPs is essentially random between linker and core regions in well-positioned nucleosomes (46). These results indicate that higher-order structural features of chromatin (*e.g.*, frequently interacting regions and super-enhancers) play a more dominant role in governing repair rates of CPDs and (6–4)PPs in chromatin [see (67)].

An alternative pathway that can lead to mutagenicity by CPDs is deamination. This hydrolytic process converts cytosine (C) or 5-methyl-Cytosine (^m^C) to uracil or thymine, respectively, making deamination a likely contributor to the mutagenic properties of C- containing CPDs (61). Taylor and colleagues reported that the rotational position of T^m^CG CPDs on the histone surface alters the rate of ^m^C to T deamination by as much as 12-fold (68, 69). In addition, they found that the deamination rates of CPDs at TCG sites in a stably positioned nucleosome within HeLa cells were slower for a CPD located at an intermediate rotational position compared to outward-facing positions (69). Thus, TCG sites in CPDs undergo deamination *in situ* and nucleosomes modulate both their formation and rate of deamination, events that likely contribute to the UV mutational spectrum in cells. Recently, Pfeifer and colleagues at the Van Andel Institute mapped cytosine deamination throughout the human genome, using a genome-wide method known as circle-damage-seq (70). It will be interesting to determine whether similar changes in deamination rates in nucleosomes occur across the human genome in cells.

### DNA damage in transcription factor binding sites

Modulation of UV photoproducts in DNA by protein binding was first demonstrated in the lac repressor complex of *Escherichia coli* lac operator DNA (71). Becker and Wang used a chemical method to cleave DNA at UV photoproducts to demonstrate repression or enhancement of these lesions in the UV-irradiated repressor-bound DNA, relative to UV-irradiated naked DNA, at or near the lac repressor binding sequence. This method was also used with UV-irradiated yeast to reveal transcription-dependent changes in the levels of UV-induced lesions in the control region of the GAL1-GAL10 genes (72). Later, T4 endo V cleavage at CPDs was used in combination with ligation-mediated PCR to quantitatively measure the level of CPDs in specific protein-DNA complexes (63). This technique revealed modulation of CPDs in promoter regions of several genes in intact human cells, including *c-jun, cfos*, and *PCNA* (73). Thus, modulation of UV photoproducts by TF binding appeared to be a wide-ranging phenomenon in chromatin.

**Figure 6:**
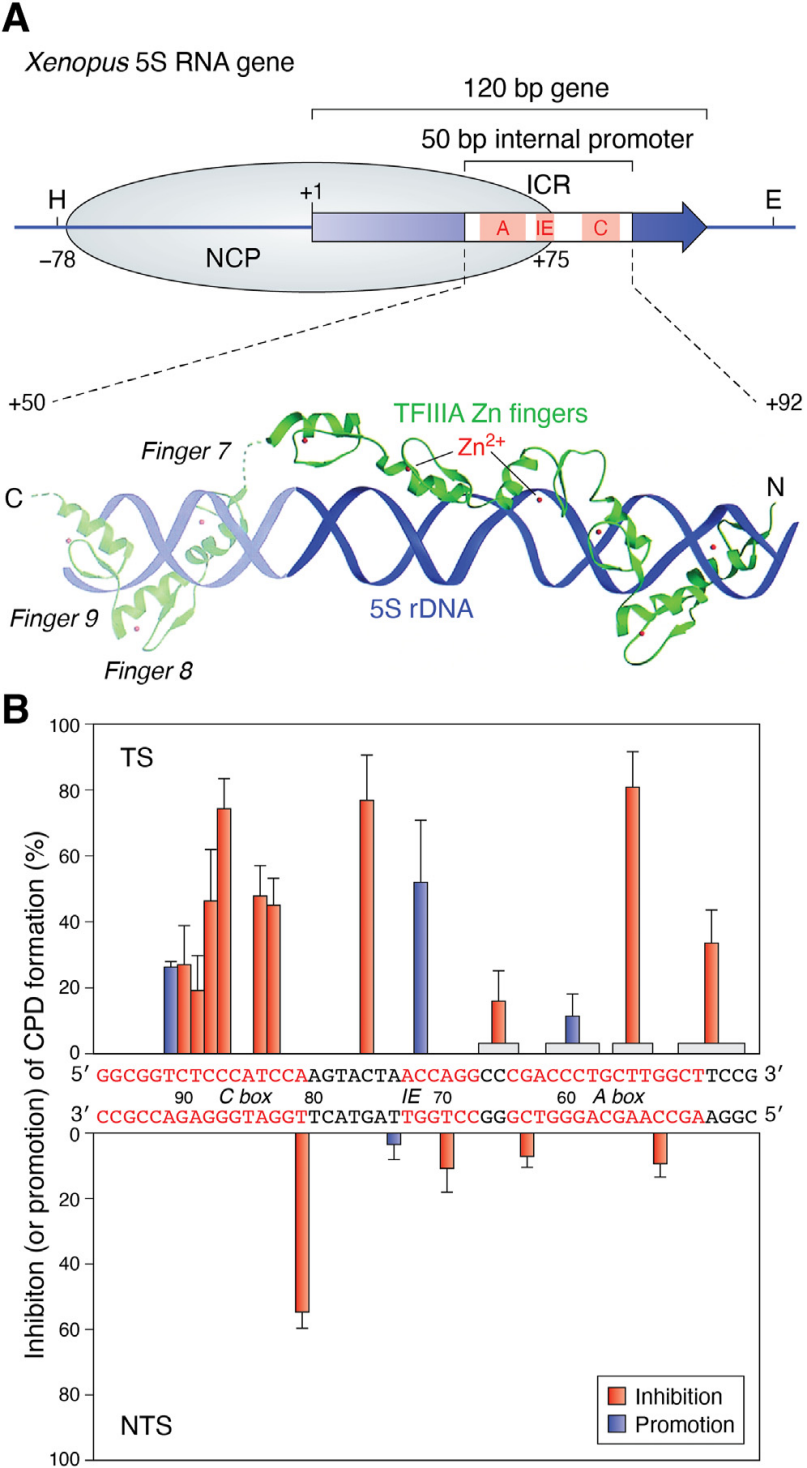
**Modulaion of UV damage by transcription factor binding.***A*, *Top*, schematic of *Xenopus* 5S RNA gene-TFIIIA complex. The 120 bp gene is denoted by a *solid blue arrow* and the 50 bp internal promoter is denoted by an *open box*. The main position of an NCP is denoted by the *light blue oval* and the *dyad center* is at approx. position −3 with respect to the transcription start (+l). The locations of the internal promoter elements A-box, intermediate element (lE), and C-box are denoted by light red boxes. (A-Bottom) Model for TFIIIA zinc fingers bound to 5S rDNA (modified from (76)). The DNA double helix (*purple ribbons*), TFIIIA zinc fingers (*green ribbons*), and Zn(II) ions (*red dots*) are shown. *B*, modulation of CPDs by TFIIIA binding. Inhibition (or enhancement) of CPD formation by TFIIIA binding at different sites, relative to naked DNA, are represented by *red or blue bars*, respectively. Locations of the C-Box, IE, and A-Box are denoted by red bases on each strand. Average values for pyrimidine tracts are denoted by horizontal (*light grey*) boxes over the top strand (modified from (75)).

To study UV photoproduct modulation at TF binding sites, the TFIIIA-5S ribosomal RNA gene (rDNA) complex, a locus containing multiple transcription units, has been a useful model system (74, 75). The TFIIIA protein contains nine tandemly repeated zinc finger motifs that bind to an internal control region (ICR) of 5S rDNA, which is an ∼50 bp segment within the transcription unit (Fig. 6*A*, top) (76). The ICR has three subdomains of protein-binding: an A-box from +50 to +64, an intermediate element (IE) from +67 to +72, and a C-box from +80 to +97 (for review, see (77)). The N-terminal zinc fingers (zfs one–3) of TFIIIA strongly bind the C-box, the C-terminal fingers (zfs seven–9) strongly bind the A-box, and the three middle zinc fingers interact with the IE sequence (Fig. 6*A*, bottom).

The effect of TFIIIA binding on UV photoproduct formation was studied in detail in the *X. borealis* 5S rRNA gene, and it was found to modulate photoproducts primarily in the transcribed strand (TS) of the 5S gene (75). This agrees with structural studies of the TFIIIA-5S rDNA complex, showing strong contacts between TFIIIA and the TS (77). Furthermore, the modulation pattern is not uniform within the template strand (Fig. 6*B*). There is strong inhibition of CPD formation at four sites in the C-box, the most important region for accurate TFIIIA binding, whereas only one CPD site is strongly inhibited in the A-box (Fig. 6*B*).

Interestingly, *enhanced* CPD formation is observed at one site in the TS of the IE region when TFIIIA is bound (Fig. 6*B*). This region binds the three middle zinc fingers of TFIIIA differently than the binding of the other zinc fingers (77). The N- and C-terminal fingers wrap around DNA within the major groove, while the three middle zinc fingers (zfs four–6) interact almost parallel to the helix axis (Fig. 6*B*). The enhanced CPD formation in the IE region suggests that the interaction of TFIIIA with 5S rDNA may cause bending that facilitates CPD formation (see above). Indeed, TFIIIA was shown to induce a substantial distortion in the structure of 5S rDNA upon binding the ICR (78).

### Impact of DNA damage modulation on mutation rates

**Figure 7:**
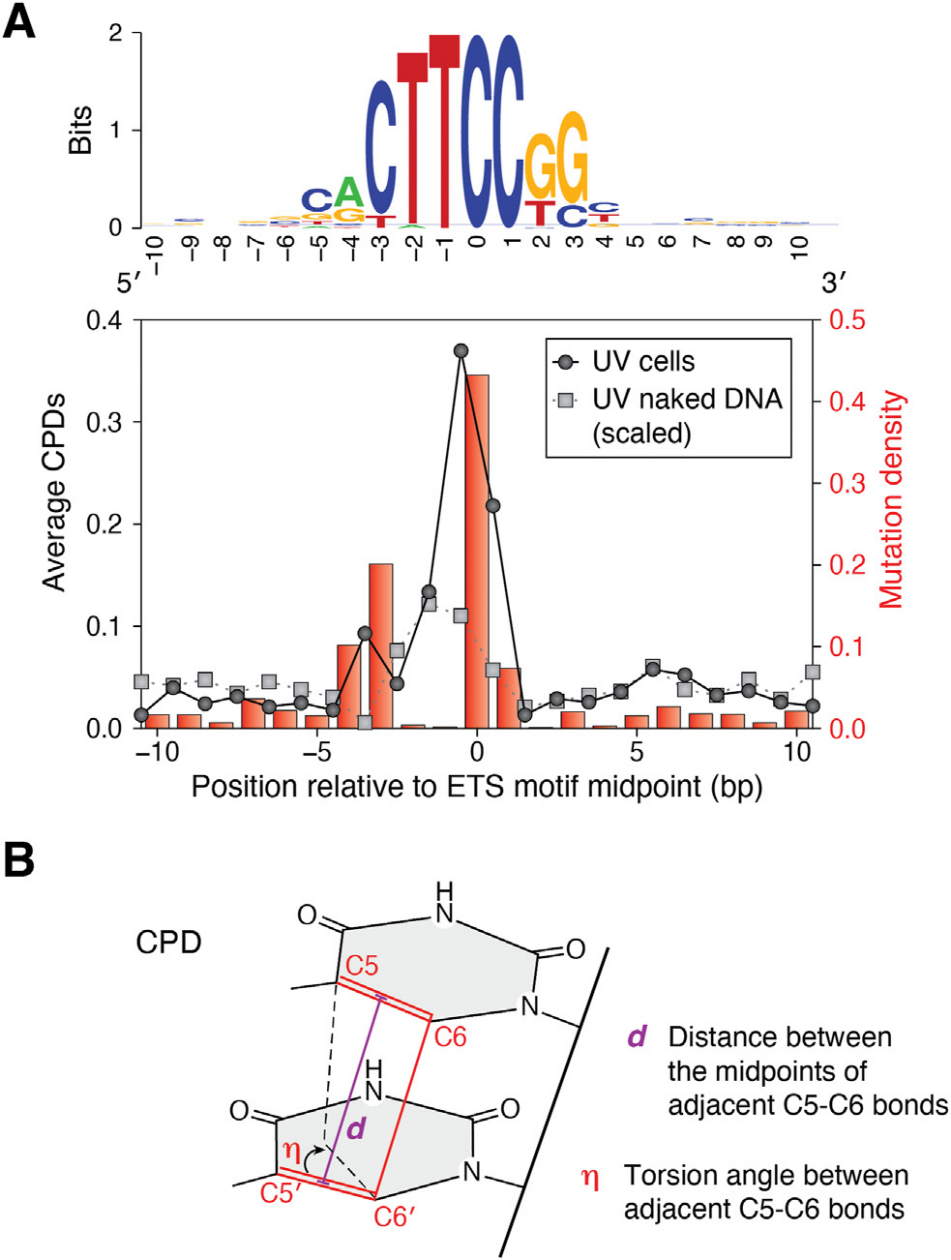
**CPDs and mutations are elevated at specific locations in ETS binding sites**. *A*, UV-induced CPD formation and mutation density at active, promoter-proximal TFBS for the ETS TFs ELK4, ETS1, and GABPA. *Upper panel*: Information content of 1279 DNA sequences for the aligned TFBS, matching the known ETS consensus binding motif. *Lower panel*: Mutation density plots for 183 melanoma tumors relative to average CPD levels following UV-irradiation of human fibroblast cells () or isolated DNA *in vitro* () (modified from (79)). See text for details. *B*, schematic of key structural parameters affecting CPD formation. The distance (***d***) between the midpoints of adjacent C5–C6 bonds, and the torsion angle (***η***) between the adjacent C5–C6 bonds (modified from (79)).

In UV-irradiated human fibroblasts, genome-wide damage mapping has shown that CPD formation is generally elevated at active transcription factor binding sites (TFBS) (79). Among 82 different TFs analyzed, two classes showed a striking induction of CPD formation at their binding sites: the ETS (E26 Transformation-specific) TF family and NFYA/B (Nuclear Factor-Y) family. The NFYA/B TF primarily induced CPDs at a TT dinucleotide in the TFBS that is not typically mutagenic in human cells. ETS binding sites, however, revealed unique damage-mutation hotspots, with up to a 16-fold increase in CPD formation and over a 100-fold increase in mutation density in melanoma (79, 80) (Fig. 7*A*). Indeed, at certain ETS binding sites (*e.g.*, RPL13 gene promoter) a single low dose of UVB treatment (20 J/m^2^) induces mutations in the RPL13A ETS motif of cultured human cells (80). As the occurrence of ETS mutation hotspots was independent of both NER pathways, the increased CPD formation at ETS binding sites was likely the major factor in the elevation of mutation rates (80).

The molecular mechanism for the extreme UV susceptibility of ETS1-bound DNA was also investigated by Mao and coworkers (79). Analysis of 13 structures of ETS1 bound to various DNA sequences revealed the distance (*d*) between the C5–C6 double bonds of adjacent pyrimidines and the torsion angle (*η*) between these bonds are favorable for CPD formation (Fig. 7*B*). Furthermore, *isolated* ETS1 protein binding directly stimulated CPD formation in the TFBS after UV irradiation *in vitro*, and this was likely due to the protein binding-induced changes in DNA structure (*i.e.*, *d* and *η* values) that favor CPD formation (79). Also, a similar structural mechanism is likely responsible for CPD induction at a specific position in the DNA-binding sites of the insulator protein CTCF (81). Notably, the location of CPD induction in the CTCF binding sites coincides with mutation hotspots in skin cancers such as melanoma (81).

## Alteration of chromatin structure by DNA damage

### Disruption of the nucleosome and higher order chromatin structure

Early studies, using a DNA supercoil assay to estimate nucleosome density, found that only about half the number of nucleosomes can be reconstituted onto closed circular plasmid DNA following irradiation with up to 3 kJ/m^2^ UV light (82). On the other hand, reduced yields in nucleosome assembly were not observed when nucleosomes were reconstituted with a portion of the yeast DED1 promoter (called HISAT), following irradiation with up to 4 kJ/m^2^ UV light (83). Competitive reconstitution experiments, however, indicated that the nucleosome formation energy (ΔG) increases on linear 5S rDNA fragments, following UV irradiation with 0.5 or 2.5 kJ/m^2^ (84). It was found that ΔG increases from that of undamaged DNA (*i.e.*, ΔΔG) by ∼ 0.2 kcal/mol for a single CPD lesion (Table 1), reflecting a higher energy barrier for CPD-containing DNA to form nucleosomes. Thus, UV lesions appear to reduce the stability of nucleosomes formed on linear DNA, and the magnitude of this effect likely depends on the DNA sequence.

**Table 1:** Relative affinities of nucleosome positioning sequences

+/− DNA damage^a^	
DNA sequence	ΔΔG°, kcal mol^−1^
Bulk chicken genomic DNA	+0.55 ± 0.03^b^
Chemically synthetic random DNA	+0.5 ± 0.13^b^
UV damaged 5S rDNA	+0.2^c^
5S rDNA	+0.00
BPDE damaged 5S rDNA	−0.3^c^
Highest affinity mouse genomic DNA	−1.82 ± 0.29^b^
Widom 601 Positioning Sequence	−2.9 ± 0.14^b^

a ΔΔG° values (mean ± standard error) are for competitive reconstitution experiments, relative to 5S rDNA.

b See (43, 243) for details.

c Values are projected for an average of one lesion/146 bp 5S rDNA from linear fits to the data in Figure 5 of ref. (84).

Mann and coworkers went on to show that nucleosome formation was *enhanced* (ΔΔG ≈ −0.3 kcal/mol) when 5S rDNA was damaged with the polycyclic aromatic hydrocarbon (+/−)- *anti*-benzo[*a*]pyrene diol epoxide (BPDE) prior to NCP formation (Table 1) (84). The authors hypothesized that (±)-*trans*-BPDE adducts promote a favorable DNA conformation for NCP formation since (a) the major DNA adduct of racemic BPDE (∼90%) is the N^2^ of guanine (85), (b) GC-rich sequences are mainly positioned away from the histone surface (49), and (c) the minor groove width is expanded with (±)-*trans*-BPDE adducts (86). These observations were extended by Broyde and coworkers at New York University, using molecular dynamics to show that the potent tumorigen dibenzo[*a,l*]pyrene also stabilizes NCPs (87). Additionally, Broyde’s group showed that the (+)-*cis- anti*-B[*a*]P-dG adduct is more destabilizing than the smaller, more constrained 5′,8-cyclo-2′-dG lesions in NCPs, indicating that DNA repair enzymes have more access to the bulky, nucleosome destabilizing (+)-*cis-anti*-B[*a*]P-dG lesion (88).

The question of whether the rotational setting of nucleosome DNA is affected by DNA damage has been studied directly in only a few cases. In an early study, it was found that the rotational setting of *mixed-sequence* DNA changes to accommodate CPDs during nucleosome reconstitution (89). On the other hand, irradiation of the yeast HISAT DNA in *preformed* nucleosomes with 4 kJ/m^2^ of UVC did not alter the rotational setting (83), indicating that this particular nucleosome can accommodate the DNA distortion associated with CPD formation. This result is in accordance with the crystal structure of an isolated CPD-containing NCP reconstituted with a palindromic nucleosome positioning sequence (NPS) having two CPDs introduced at symmetric sites (65).

Using an alternative approach, Smerdon’s group showed that when cyclobutane thymine dimers (CTDs) were incorporated at each position of a complete turn of the DNA helix near the dyad axis of a strong NPS, these UV lesions did not change the rotational setting of the DNA, regardless of their position (90). Even NCPs containing two CTDs separated by ∼1/2 turn of the DNA helix maintained the rotational setting imposed by the NPS. Moreover, the deletion of small segments of the NPS to shift the rotational setting of the DNA caused the two CTDs to shift to newly imposed rotational settings. Smerdon and coworkers performed a series of gel-shift analyses to show that one CTD destabilizes histone-DNA interactions by 0.6 ±0.12 kJ/mol or 1.1 ± 0.2 kJ/mol when facing **Out** (toward the solvent) or **In** (toward the histone surface), respectively (90). This indicates that the ∼ 0.5 kJ/mol energy penalty for a *buried* CTD is not enough to change the rotational setting of sequences with strong rotational preference in NCPs. In the case of two CTDs ∼1/2 turn apart, they found that DNA-histone interactions are destabilized by 1.6 ± 0.3 kJ/mol, or close to the sum of the change in free energy penalties for each lesion alone (90). Thus, the CTD sites appear to act almost independently, consistent with a *localized* disruption in DNA-histone interactions at each site. Also, these changes in free energy are similar to values reported previously for randomly positioned CPD lesions within 5S rDNA NCPs (84). It is important to note that, although these free energy differences are small, they are significant for the majority of genomic DNA sequences where the rotational setting in NCPs is supported by ΔΔG values closer to 1 kJ/mol (91) (see also Table 1).

The effects of DNA damage on the rotational setting in nucleosomes were also examined for cisplatin-induced 1,2-days(GpG) and 1,3-days(GpTpG) intrastrand cross-links by Lippard and colleagues at MIT (92). These lesions were shown to change the DNA rotational setting of a moderately robust NPS by constraining the Pt adduct orientation to face inward toward the histone core. Thus, it appears that some nucleosomes (*e.g.*, with certain DNA sequences and NCP positioning power) can tolerate the distortions of some DNA destabilizing lesions and supersede the energy penalty of having these lesions at certain sites.

Damage to DNA can also influence nucleosome unwrapping dynamics [reviewed in (93)]. For example, UV-induced photolesions promote increased DNA unwrapping from the histone octamer (94). This increased unwrapping activity was detected even when NCPs contained just one CPD or (6–4)PP lesion at a single site in the NCP DNA. Unexpectedly, the CPD lesion was more efficient in driving NCP unwrapping than the (6–4)PP when each was inserted at SHL1.5 (15 bp from the dyad center). As (6–4)PPs produce greater helix distortion than CPDs in identical duplexes (95), the large kinking angle around a (6–4)PP at SHL1.5 may restrict the DNA curvature in NCPs and reduce the rate of nucleosome unwrapping. These results raise the possibility for increased “intrinsic exposure” of nucleosome-associated DNA lesions in chromatin to DNA repair proteins.

Studies on the effects of DNA lesions on higher-order chromatin structures are not as straightforward, as these structures are heterogeneous and less well-defined (96). Early studies relied on low-resolution methods to obtain evidence that DNA damage may disrupt higher-order chromatin packaging. Hittelman at the University of Texas used “premature chromatin condensation,” obtained by fusing interphase and mitotic cell nuclei, to show that large sections of chromatin are stably decondensed in UV-irradiated cells (97). These decondensed regions of chromatin rapidly become visible in a traditional light microscope. However, it was likely that this decondensation resulted from DNA repair processing rather than a direct physical distortion of higher-order chromatin by UV damage. On the other hand, differential scanning calorimetry revealed that certain anticancer drugs directly altered the DNA melting profile of chromatin in intact nuclei (98). Finally, physical studies on the folding of polynucleosomes *in vitro* indicated that even large doses of trimethylpsoralen cross-links or UV photoproducts are accommodated during salt-induced polynucleosome condensation (99). Therefore, early studies found that direct physical alterations by some DNA lesions in chromatin appeared to be much more subtle compared to the chromatin processing response by repair of these lesions (see Alteration of chromatin structure during DNA excision repair).

### Disruption of transcription factor binding sites

The consequences of DNA damage on TF-DNA interactions have been the focus of numerous studies in the past. It was shown that DNA adducts can affect TF binding, but the degree of alteration depends on both the type of adduct formed and the sequence of the TFBS. For example, high-mobility group protein HMGl and human upstream binding factor (hUBF) bind mixed-sequence DNAs containing cisplatin adducts with high affinity (100, 101). These results were followed by experiments with specific TFBS containing cisplatin adducts, which led to the observation that these high-affinity DNA adducts can act as “decoy binding sites” for TFs and suppress DNA repair by shielding the DNA lesions (102). In addition, high-affinity binding occurred with the TF Spl when BPDE adducts are present in *nontarget* DNA sequences (103). Surprisingly, it was later found that BPDE adducts *within* the TFBSs of Spl and AP-1 inhibited the binding of these two proteins (104, 105).

It was also shown that the alkylation of DNA can inhibit TF binding, including NF-*k*B, Spl, OTF-1, and AP2 (106, 107). Also, CPDs incorporated at specific sites of oligonucleotides containing the recognition sequences of E2F, NF-Y, AP-1, NF*k*B, and p53 strongly inhibit the binding of these TFs to their cognate TFBSs (108). Moreover, UV damage can inhibit the binding of TFIIIA to 5S rDNA (109), and irradiation of the TFIIIA/5S rDNA complex displaces the TFIIIA protein (75). These latter results indicate that the TFIIIA-5S rDNA complex is unable to accommodate UV photoproducts at most sites. Therefore, binding of a variety of TFs is inhibited (or enhanced) by both DNA chemical adducts and UV photoproducts, indicating that DNA lesions can alter gene regulation and have consequential effects on physiological functions such as stress responses and disease progression. As discussed earlier, this may reflect conformational changes in the TFBS after lesion formation where the lesion structure is more (or less) compatible with TF-DNA complex formation.

It is known that TFs can facilitate DNA repair *via* transcriptional regulation of specific target genes encoding DNA repair proteins in the DDR. More recently, it was revealed that TFs may also be DNA repair components acting *directly* at DNA lesions in a transcription-independent fashion [*e.g.*, see (110)]. Recruitment of TFs to DNA lesions (*e.g.*, by binding to specialized DNA repair proteins) can directly regulate DNA repair. Thus, TFs can facilitate the DNA repair process by allowing for efficient chromatin remodeling and access to DNA repair machinery. Unlike transcriptional regulation, this recruitment of TFs to DNA lesions appears to occur in a DNA sequence-independent fashion, possibly by changing the chromatin landscape from the undamaged state.

## Regulation of DNA excision repair in chromatin

### Nucleotide excision repair in nucleosomes

**Figure 8:**
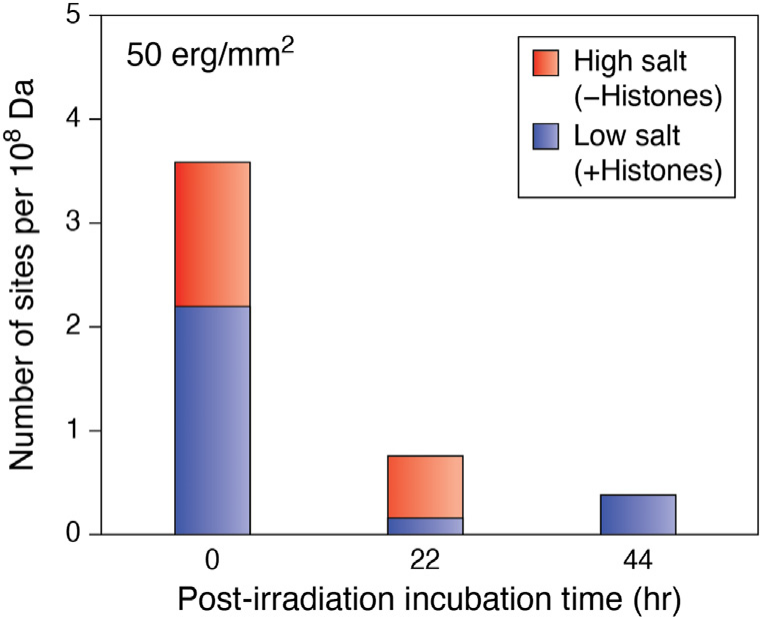
**Removal of CPDs from DNA of NHFs irradiated with UV light.** Graph shows *M. luteus* endonuclease-sensitive sites (ESS) in permeabilized WI-38 cells exposed by low salt treatment of chromatin (*blue bars*) and additional ESS exposed by high salt treatment (*red bars*) (Modified from Figure 1*A* of (111)).

One of the first studies on DNA repair in chromatin was by Wilkins and Hart at Oak Ridge National Laboratory who examined the preferential repair of UV-damaged DNA in normal human fibroblasts (NHF) (111). They reported that, after low fluences of UVC light, between 25% and 50% of the total CPDs (detected as endonuclease-sensitive sites, or ESS) in human chromatin was “unmasked” by high salt treatment, and this fraction persisted for at least 44 h (Fig. 8, solid bars). They concluded that CPDs, and possibly other UV photoproducts, “persist in tracts of DNA which are rendered refractory to excision repair by a 'mask' of protein” (111). Although this study was performed before the discovery of the nucleosome, the insightful conclusion by Wilkins and Hart was a foreshadowing of the results to come.

**Figure 9:**
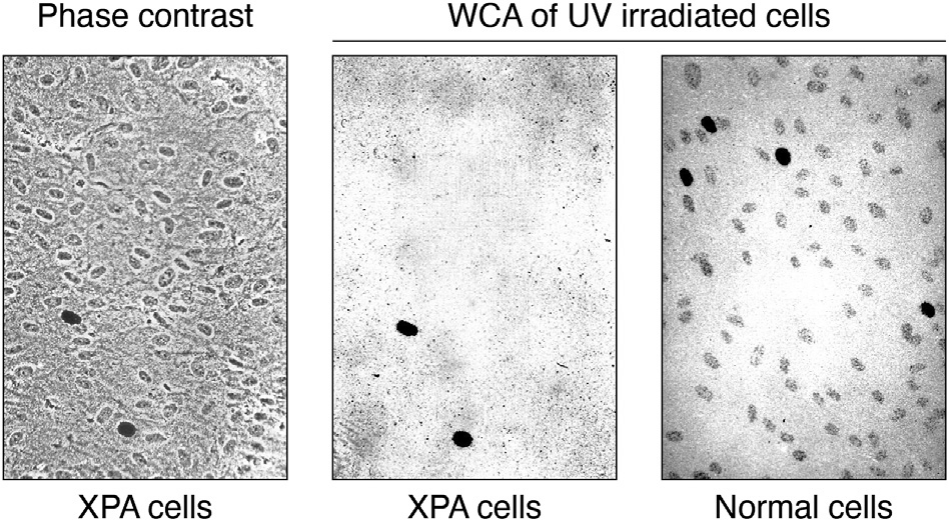
**Whole-cell autoradiography (WCA) of UV-irradiated human cells.** Normal human and *xeroderma pigmentosum* (complementation group *A*) skin fibroblasts were grown to confluence, treated with 10 mM hydroxyurea, exposed to UV light, and labeled with [^3^H]dThd (K. Sidik and M. J. Smerdon, *unpublished results*). The grains/nucleus (*e.g.*, *right hand panel*) in nondividing cells (*i.e.* cells not totally darkened by [^3^H]dThd incorporation) are a measure of NER synthesis activity. For details, see (241).

After the discovery of the nucleosome, studies on DNA repair in chromatin started to appear and focused on the distribution of NER *synthesis* in nucleosome-loaded DNA after treatment with different DNA-damaging agents (112, 113, 114). This was the preferred NER activity to measure since repair patches in cultured cells could be labeled with high specific activity [^3^H]dThd after treatment of nonreplicating (or replication suppressed) cells with DNA damaging agents. For example, whole cell autoradiography of cultured cells, labeled with [^3^H]dThd, was an important technique for measuring DNA repair synthesis in chromatin over the past 50 years (Fig. 9). This technique was used by James Cleaver at the University of California San Francisco in his seminal study demonstrating UV-irradiated cells from patients with the cancer-prone disease *xeroderma pigmentosum* (XP) are deficient in NER synthesis (115) (Fig. 9, middle panel).

**Figure 10:**
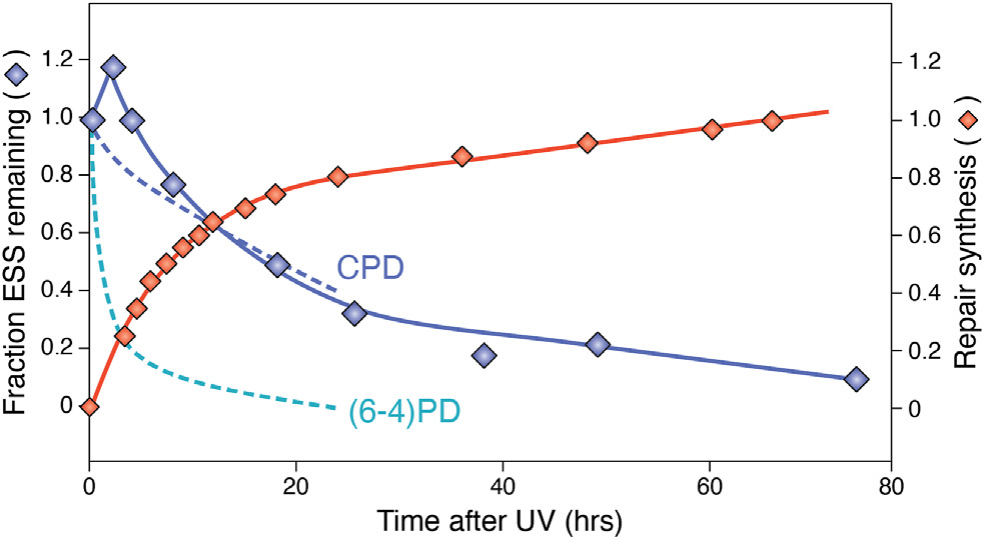
**Repair of UV damage to DNA in confluent NHF.** T4 endonuclease-sensitive sites (ESS) were determined for total DNA from confluent NHF, irradiated with 12 J/m^2^ UVC light, and incubated for times shown. Values () denote the fraction of ESS remaining relative to zero repair time. Repair synthesis incorporation of [^3^H]dThd () was determined for these same cells. The time course of CPD and (6–4)PP removal from NCPs (*upper* and *lower dashed lines*, respectively) isolated from these cells is also shown. For details, see (6).

Virtually all the early studies found enhanced NER synthesis within *micrococcal* nuclease (MNase) accessible DNA in chromatin (112, 113, 114, 116, 117). Lieberman and colleagues at Washington University showed that the nuclease-resistant DNA in NCPs was especially low in UV-induced NER synthesis (114, 116, 117). These findings spawned the notion of “preferential repair synthesis in nuclease-sensitive regions of chromatin during fast repair” and the “underrepresentation of fast-repair synthesis in nuclease-resistant regions” (114) (see Fig. 10, open diamonds).

During the 1980s and 1990s, the distribution of NER synthesis *within* nucleosomes was examined extensively, especially in UV-irradiated NHFs [reviewed in (6)]. It was established that overall repair synthesis, following different continuous labeling times after UV irradiation, occurred in two phases in these cells: an early rapid phase and a prolonged slow phase (114) (Fig. 10, open diamonds). During the rapid phase, the majority of (6–4)PP are removed from NHF DNA, while a significant fraction of CPDs remains until the slow phase of repair (45) (Fig. 10, dotted lines). Furthermore, during early repair times, NER synthesis is nonuniform in nucleosomes, having a strong bias toward the 5′ ends of NCP DNA (79, 118), a result supported by recent observations of asymmetric removal of CPDs in nucleosomes and strand polarity of somatic mutations (119).

Repair synthesis occurring during late times after UV irradiation (>24 h) was found to be more randomly distributed in NCPs (114, 118). In addition, although these late incorporated repair patches appeared to be somewhat shorter than those incorporated during the early rapid phase (118, 120), recent XR-Seq data indicates that the average length of the excised CPD-containing oligomer remains similar even after long repair times (56). These findings indicated that UV photoproducts are either more accessible to NER enzymes in the 5′ ends of NCP DNA or UV photoproducts form preferentially in these regions.

These possibilities were tested using a T4 Polymerase-exonuclease blockage assay (47) to map the CPD distribution in NCP DNA of NHF cells during the early and late NER phases (120). Little change was observed in the periodic pattern during the fast repair phase, indicating that this phase does not reflect preferential repair in the 5′ ends of NCPs. This also inferred that CPDs are removed at ∼ equal rates by NER from the inner and outer facing sides of the DNA helix in NCPs. On the other hand, it was observed that CPDs form preferentially in the 5′ ends of NCP DNA, showing a bias that accounted for much of the nonuniform distribution of repair patches observed during the early rapid NER phase (120). Therefore, preferential UV damage near the ends of NCP DNA seemed to be the most likely explanation for the nonuniform distribution of repair synthesis within NCP DNA. Consequently, other factors were most likely responsible for the two NER phases in human cells (Fig. 3).

**Figure 11:**
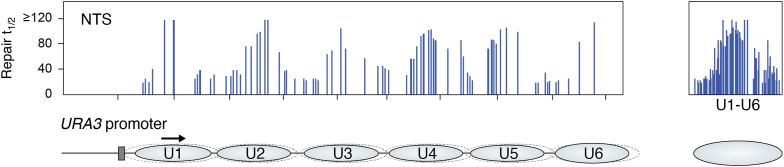
**Repair of UV photoproducts in the *URA3* gene of yeast (Modified from**Figure 1A**of** (8)**).** (*Left panel*) The time, in min, to remove 50% of the photoproducts (t_1/2_) at each lesion site (*vertical lines*). (*Right panel*) Superposition of all 50% repair times measured in the NTS of six *URA3* nucleosomes (U1-U6). Schematic denotes the six nucleosomes in the *URA3* gene and their main positions (*light blue ovals*, *solid line*) along with their minor positions (*dashed lines*). *Arrow* denotes the direction of transcription. For details, see (8).

As will be discussed in Alteration of chromatin structure during DNA excision repair, DNA repair patches are inserted *after* nucleosome rearrangement (or unfolding) in human cells (6, 117). Therefore, it was not possible to determine if nucleosomes modulate DNA repair during the early, rapid phase by simply examining the repair patch location in chromatin. However, convincing evidence for the modulation of NER by nucleosomes came from studies by Thoma and colleagues, at the Swiss Federal Institute of Technology (ETH), who examined the repair of the nontranscribed strand (NTS) of the *URA3* gene in yeast genomic chromatin and in isolated minichromosomes (8, 121). Using a primer extension technique, the UV photoproduct removal (primarily CPDs) occurs more rapidly in linker DNA and toward the 5′ ends of positioned NCPs in the *URA3* gene of *Saccharomyces cerevisiae* (Fig. 11). Slow removal of photoproducts occurred within the internal protected regions (near the dyad axes) of the six NCPs present (Fig. 11, boxed panel), and the repair efficiencies (*i.e.* “50% repair times”) within these NCPs correlated well with the efficiencies of cutting by DNase I (121). Therefore, this study provided compelling quantitative evidence that, in the absence of transcription, NER in yeast is indeed modulated by DNA packaging in nucleosomes.

A second contributor to NER synthesis during the early rapid repair phase is the removal of (6–4)PPs (Fig. 10, lower dotted line). Although both CPDs and (6–4)PPs are removed by NER, the overall rate of repair of (6–4)PPs in genomic DNA is more rapid than CPDs (122). Given the distribution of (6–4)PPs in chromatin (see Modulation of the distribution and yield of DNA damage in chromatin), their rapid repair could result, in part, from being more accessible to repair enzymes than CPDs. This possibility was examined in isolated NCP DNA from UV-irradiated NHF cells (123). Using radio immunoassays for detection of the two different UV photoproducts, it was observed that (6–4)PPs are removed faster than CPDs, even from NCPs in intact NHF (Fig. 10, compare dotted lines). Thus, the majority of (6–4)PPs are removed during the early rapid phase of repair in human cells (Fig. 10, compare lower dotted line and open diamonds), which accounts for up to half of the repair synthesis observed during this period.

The effect of the rotational setting of DNA on CPD removal from the histone surface in NCPs (see Fig. 2, *A*–*C*) was also examined, using the NER activity of *Xenopus* oocyte nuclear extracts (90). In these studies, the Smerdon group found that NER rates (expressed as %CTDs removed per hour) were only 2 to 3 times lower in nucleosomes than in naked DNA. Importantly, the NER rate changed by only about 1.5-fold for CTDs facing **Out** compared to those facing **In** toward the histone surface (90). Thus, in the presence of *Xenopus* nuclear extracts, the rotational orientation of CTDs on NCPs has surprisingly little effect on the rate of NER. These results indicated that nucleosome dynamics and/or chromatin remodeling activity (present in the nuclear extracts) were facilitating NER proteins in gaining access to UV damage in nucleosomes.

Importantly, Matsumoto’s group has recently found that the UV-damaged DNA-binding protein (UV-DDB) can bind occluded (6–4)PPs in strongly positioned nucleosomes by changing the predominant rotational orientation of the NCP DNA (124).

Finally, an additional pathway, not found in humans, for the repair of CPDs exists in many organisms that involve direct photoreversal of the cyclobutane bond between pyrimidines (57). This activity is carried out by a single enzyme, called photolyase, and is present in a variety of different eukaryotic organisms, including yeast. Therefore, the question arose as to whether the activity of photolyase is also modulated by nucleosome structure. Once again, the Thoma group used yeast strains containing minichromosomes with well-characterized structures to show that nucleosomes indeed modulate photolyase repair (8, 125). They found that the photolyase activity in yeast cells rapidly repairs CPDs in nucleosome linker DNA and nonnucleosome regions of the minichromosomes. Furthermore, in contrast to NER, repair of the TS of an inducible gene by photolyase was *inhibited* by RNA Pol II transcription, showing a lack of transcription-coupled photoreactivation repair (125) (see Regulation of DNA excision repair in transcriptionally active chromatin). These findings suggested that RNA Pol II blocks the action of photolyase at CPDs by inhibiting photoproduct accessibility to the enzyme (reviewed in (8)). Thus, photoreactivation repair is more sensitive to nucleosome packaging than NER in yeast chromatin and does not appear to be coupled to transcription.

### Base excision repair in nucleosomes

The effect of nucleosome formation on BER has been examined extensively over the last 2 decades [see reviews by (7, 126)]. The first reports examined BER activities on chromatin substrates *in vitro* using isolated BER enzymes (*e.g.*, human uracil DNA glycosylases (UDG, UNG2 and SMUG1), APE1 and Pol ß) and NCPs with uracil at defined locations. One study used a moderate NPS (*Lytechinus variegatus* 5S rDNA) with uracil residues at sites more than two or five helical turns from the dyad center (127). The other study used a strong NPS consisting of a glucocorticoid receptor element (or GRE) bracketed by multiple, positioned TG-motifs with uracil residues located ½ turn 3′ or ½ turn 5′ from the dyad center (128). Both studies found a significant reduction in the activities of BER enzymes when the phosphate backbone of the uracil-containing DNA was facing toward the histones. However, Nilsen and coworkers found that the efficiency of uracil excision from the 5S rDNA NCP was essentially uniform along the DNA, irrespective of rotational position (127). In contrast, Beard and coworkers (128) found a significant difference in uracil excision activity between the two different rotational settings in the TG-NCP’s, being two- to threefold lower for uracil facing **In** toward the histones (see Fig. 2, *A*–*C*).

Together, these two studies revealed a critical role for nucleosome stability in the recognition of DNA damage and completion of BER. The 5S rDNA is less constrained on the histone surface than the TG-GRE-TG motif (129), and has multiple translational settings (130), allowing more torsional and translational flexibility. The flexibility of DNA *along the helix axis* was addressed in both studies by following the synthesis of Pol ß (after cleavage by UDG and APE1). The lack of Pol ß synthesis observed by Beard *et al*. (128), and the partial inhibition of Pol ß synthesis observed by Nilsen *et al*. (127) again likely reflects the differences in NCP stability between the two nucleosome substrates as well as the difference in uracil locations within the NCPs.

Hayes and colleagues at the University of Rochester, as well as the Smerdon group, studied the effect of rotational and translational locations of uracil in more detail. While the cleavage rate by either *E. coli-* or human-UDG on U-**Out** NCPs was found to be moderately lower than that of naked DNA (*e.g.*, 3–6 fold for *E. coli* UDG), cleavage rates for U-**In** and U-**Mid** NCPs were significantly reduced (*e.g.*, >1000-fold for *E. coli* UDG) (131). Furthermore, the Hayes group showed that *E. coli* UDG activity on DNA just *outside* the NCP region was similar to that of naked DNA (131). They also showed that the association of linker histone (H1) significantly reduced the activity of *E. coli* UDG at sites where the globular domain of H1 binds to nucleosomes. Additionally, the Smerdon group showed that crosslinking of U-**In** DNA to histones in NCPs yielded a marked reduction in human UDG cleavage rate but, surprisingly, produced an *increased* cleavage rate in U-**Out** NCPs (132). The Smerdon group also found that the next enzyme in the BER pathway, APE1, stimulated the activity of human UDG in U-**Out** NCPs, suggesting that UDG and APE1 interact on the surface of histones in orientations accessible to UDG. Thus, the activity of UDG may require “trapping” transiently exposed states arising from the rotational dynamics of DNA on histones.

The effect of uracil positions in NCPs on the first three activities in BER was also examined by Rodriguez and Smerdon (133). In agreement with prior studies, which used different NPSs (131, 132), the removal of DNA lesions was greatly dependent on their rotational and translational positioning in 601 NCPs (Table 1). Uracils with inwardly oriented minor grooves located farther away from the dyad center of 601 NCPs were more accessible to UDG/APE1 than those located near the dyad. In addition, the translational positioning of outwardly oriented single nucleotide gaps was the key factor driving Pol ß gap-filling activity (133). For example, an outwardly oriented gap near the DNA ends yielded a threefold higher gap-filling activity compared to gaps with the same rotational orientation near the dyad center. Interestingly, UDG/APE1 efficiently removed an outwardly oriented uracil ∼1 helical turn from the NCP dyad, while Pol ß gap-filling activity was significantly inhibited at this site (133). These data suggest that hindrance at the location of a DNA lesion is dependent on the structural requirements for enzyme catalysis.

An explanation for the different substrate features of glycosylases and Pol ß may relate to the structural constraints these enzymes impose on DNA during catalysis. DNA glycosylases induce a 45° to 70° bend in the lesion-containing strand of naked DNA (reviewed in (7, 15)), while Pol β bends the strand opposite the gap by ∼90° in naked DNA (reviewed in (134)). This high degree of DNA bending may limit the ability of Pol ß to function on the NCP surface.

Alternatively, Pol β may be able to sufficiently disrupt histone-DNA contacts near a gapped site when bound to an outward facing minor groove of NCP DNA, but not when the minor groove faces inward where multiple histone-DNA contacts occur, and limited DNA unwrapping occurs(see (49, 135)).

Recently, Wilson and colleagues helped clarify the reduced Pol ß activity on nucleosomes. These investigators examined which of the Pol ß activities (5′-dRP lyase or template-directed DNA synthesis) is most affected by the rotational setting of a single nucleotide gap on the NCP surface (136). They found that different rotational orientations have little effect on the 5′-dRP lyase activity of Pol ß, whereas a strong inhibition is observed with DNA synthesis. In a separate report, Wilson and coworkers show that this strong inhibition of Pol ß gap-filling synthesis in NCPs also inhibits the productive processive searching of Pol ß for single base lesions on a nucleosome template (137). Thus, in the absence of additional factors, the stalling of BER at nucleosomes likely produces an accumulation of aborted, potentially mutagenic, intermediates in chromatin, and rearrangement of DNA at damage sites in nucleosomes is critical for ensuring the completion of BER (136, 137).

An earlier study provided insight into the role chromatin remodeling may play in promoting efficient BER in chromatin (138). The Smerdon and Wilson groups examined the catalytic activities of purified human BER enzymes on oligonucleosome arrays (containing 12 tandem repeats of a 208 bp segment of the *L. variegates* 5S rDNA) with uracil randomly incorporated at cytosine bases following treatment with sodium bisulfite. They found that, although UDG and APE1 digested G:U mismatches to completion in folded oligonucleosomes, Pol ß gap-filling synthesis was inhibited in ∼80% of the DNA in these arrays or the ∼ fraction in NCPs (138). This suggests that single-strand gaps in linker DNA are far more accessible to Pol ß in folded oligonucleosomes. Importantly, this inhibition of Pol ß synthesis in folded oligonucleosomes was removed by purified chromatin remodeling complexes ISW1 and ISW2 from yeast (138). This result indicates that chromatin remodeling may be required for the latter steps of BER in NCP domains of nucleosomes.

As discussed earlier, another feature of the polymerization step in BER is that polymerization can progress with either short patch BER, where 1 nt is inserted by Pol ß, or long patch BER, where two to ∼13 nts are inserted by either Pol ß or Pol *δ*/*ε* (4, 15). Using cell-free extracts or purified enzymes, Meas and Smerdon showed that the location of lesions in nucleosomes determines which of these sub-pathways is used (139). DNA lesions within NCPs are preferentially repaired by Pol β and there is a substantial reduction in BER synthesis beyond 1 nt (139). When Pol β was immunodepleted from the extracts, BER in nucleosomes was significantly reduced. Long patch BER occurred exclusively in linker DNA, with the extension of these repair patches ending at the edge of NCPs (139).

To this point, we have focused on the BER of uracil in DNA as this was the first nucleotide “lesion” to be studied in detail at the nucleosome level. However, the majority of spontaneously occurring DNA damage in cells is from hydrolytic and oxidative reactions with water and ROS, respectively (5). Pederson and coworkers at the University of Vermont investigated the activity of purified human glycosylases and APE1 to initiate BER at oxidative lesions [*e.g.*, Thymine glycol (Tg), tetrahydrofuran and polyunsaturated aldehydes] in nucleosomes designed with the *L. variegatus* 5S rDNA NPS. As observed with UDG/APE1 cleavage of uracil, when the minor groove of Tg residues faces **Out** on the NCP surface, the bifunctional human DNA glycosylase hNTH1 cleaves at Tg with similar efficiency as in naked DNA (140). However, APE1 does not stimulate hNTH1 activity in nucleosomes, while hNTH1 has a significant effect on APE1 activity in naked DNA (141). Furthermore, at these same concentrations, hNTH1 cleavage activity at lesions facing **In** toward the histone octamer was markedly reduced, but increased considerably at hNTH1 concentrations closer to physiologic levels in the cell. In addition, lesions facing **In** near the nucleosome edge were more efficiently processed than one located near the nucleosome dyad (140). Pederson and colleagues initially hypothesized that access to the occluded lesions facing **In** resulted from DNA unwrapping in NCPs, allowing hNTH1 to capture the Tg lesion when DNA is in the unbound state (140). However, they later performed detailed kinetic analyses with **In** facing Tg lesions at different translational settings in NCPs constructed from the Widom 601-NPS (142) and found that the rates of DNA unwrapping in NCPs are too low to account for the rates of BER in cells. Therefore, they concluded that some form of chromatin rearrangement must play an important role in efficient BER *in vivo*.

The Pederson group also studied the completion of BER in nucleosomes by probing the ability of Pol ß and LigIIIα-XRCC1 to close and ligate a 1 nt gap (143). Since DNA ligases almost completely encircle their DNA substrates (144), it is likely that LigIIIα-XRCC1 requires the disruption of at least local histone-DNA contacts in NCPs for their function. Indeed, Pederson and colleagues showed that LigIIIα-XRCC1 activity on gapped- or nicked- DNA within NCPs is critically dependent on enzyme concentration, regardless of the rotational orientation of the gap or nick (143) and this complex performs DNA nick repair after transient unwrapping of nucleosomal DNA (145).

A wider view of glycosylase activity in nucleosomes was provided by Delaney and colleagues at Brown University (146), who compared the activities of five different glycosylases in the removal of their preferred lesions from well-characterized 601 NCPs. Their results show that DNA glycosylase activity on NCPs is highly variable. Factors affecting their efficiency include the solvent accessibility and identity of the damaged base, as well as the size, structure, and catalytic mechanism of the glycosylase proteins themselves (146).

The Delaney group then examined the dependence of 8-oxoG repair by the bifunctional human DNA glycosylase hOGG1 on the transient unwrapping of NCP DNA (147, 148). They had shown that 8-oxoG, U, and εA are poorly repaired regardless of their rotational orientation in NCPs when located in the ∼20 bp region centered around the dyad axis [reviewed in (126)] and hypothesized that the diminished lesion accessibility in the dyad region may relate to the altered DNA structure of the ∼30 bp region centered at the dyad axis of NCPs (49, 149). They found that in the absence of chromatin remodelers or external cofactors, hOGG1 can actively initiate BER at positions beyond this dyad axis region and the activity appeared to be facilitated by unwrapping of DNA from the histones (148). However, initial FRET studies measured an equilibrium constant for nucleosome unwrapping of ∼0.02 to 0.1 (or nucleosomes are partially unwrapped ∼2–10% of the time), and rate constants measured for the spontaneous unwrapping of NCP DNA indicate that the mean lifetime of the partially unwrapped state is between ∼ 3 and 50 ms [reviewed in (135)]. Furthermore, unwrapping-mediated exposure to glycosylase NTH1 of an oxidative lesion near the NCP end was found to be ∼7 to 8 times per minute and fell off dramatically for lesions closer to the dyad center (142). Hence, the frequency of DNA unwrapping events that expose most lesions in NCPs *in vitro* is much lower than needed to account for the rapid repair times measured in cells, indicating that spontaneous unwrapping of nucleosome DNA *alone* is not sufficient to account for the efficient repair of all oxidative lesions *in vivo*.

Chromatin also contains histone variants, which form “variant nucleosomes” (150). These nonallelic isoforms of canonical histones can render altered nucleosome structures and provide distinct demarcations in chromosomes. For example, the human histone variant H2A.B (formerly H2A.Bbd) exchanges rapidly, compared to canonical H2A, and preferentially associates with actively transcribed genes (151). The H2A.B nucleosomes have a more extended (and relaxed) structure and are more easily transcribed than canonical nucleosomes. These nucleosomes are also more resistant to chromatin remodeling by SWI/SNF (152). Angelov and colleagues studied BER of a single 8-oxoG lesion inserted close to the dyad axis of reconstituted canonical nucleosomes and H2A.B-601 nucleosomes (153). They found that murine 8-oxoG DNA glycosylase (mOGG1), human APE1, and human Pol ß activities are strongly reduced in each of these nucleosomes, though the initial efficiency of mOGG1 cleavage was four- to 5-fold higher in the H2A.B NCP. Moreover, whereas SWI/SNF remodeling of canonical nucleosomes stimulated the processing of 8-oxoG by each of the BER factors to efficiencies similar to naked DNA, this had almost no effect on 8-oxoG removal in H2A.B nucleosomes (153). This latter observation agrees with previous studies by these authors showing that remodeling complexes SWI/SNF and ACF are unable to mobilize the H2A.B nucleosome (152).

Delaney’s group also examined the impact of substituting canonical H2A with variants H2A.Z and macroH2A on the initiation of BER in nucleosomes (126, 154). Both variants have been implicated in double-strand break repair and one of them (H2A.Z) was implicated in NER (155, 156). Excision at uracil residues by UDG and SMUG1 was evaluated using a 601 DNA population with globally distributed U:G base pairs in a wide variety of translational and rotational positions on the reconstituted NCPs (154). They observed enhanced excision in both the H2A.Z and macroH2A-containing NCPs. The U sites with reduced solution accessibility (*e.g.*, U-**In**) exhibited limited UDG activity in canonical NCPs but were more efficiently excised in H2A variant NCPs (154). The U sites with the largest increase in excision in variant NCPs are clustered in regions with differential structural features between the variants and canonical NCPs, revealing potential functions for H2A variants in promoting BER and preventing mutagenesis in chromatin (126).

Using the same experimental platform, the Delaney group also examined the impact of the H3.3 variant and the dual-variant H2A.Z/H3.3 NCPs on the initiation of BER (157). Enhanced excision of sterically occluded U by UDG and SMUG1 is observed with the H3.3 variant. For the dual-variant NCPs, the global repair profile reveals that UDG, but not SMUG1, has increased dU excision activity, highlighting the unique ways in which DNA glycosylases are impacted by histone variants.

Finally, Sczepanski and colleagues at Texas A&M University developed a “plug-and-play” approach to prepare oligonucleosome arrays with a site-specifically modified uracil (composed of 12 tandem repeats of a 147 bp segment of 601 DNA separated by 30 bp of linker) (158). The combined catalytic activities of UDG and APE1 were found to be inhibited by up to 20-fold or accelerated by up to 5-fold depending on the positioning of uracil relative to the dyad axis when compared to naked DNA and mono-NCP substrates. Furthermore, when the oligonucleosomes were incubated in the presence of a higher Mg^2+^ concentration, to condense the nucleosome array and mimic heterochromatin formation, uracil in the linker region was processed at a 5-fold increased rate relative to naked DNA. Histone H3 acetylated at lysine 18 or 27 was shown to increase or decrease, respectively, the combined activities of UDG/APE1 reflecting the potential influence of histone post-translational modifications (PTMs) on BER in chromatin (159). Thus, both NER and BER are significantly regulated by the context of the chromatin landscape.

### Regulation of excision repair and mutagenesis in higher order chromatin

Sancar and collaborators carried out genome-wide studies on NER activity in human fibroblasts using the XR-seq method to show that repair of UV damage is strongly modulated by the “global chromatin state” (56, 160). In agreement with previous studies (discussed above), these authors found that, on a genome-wide level, (a) fast repair of CPDs and (6–4)PPs occurs in open chromatin regions, and slow repair of CPDs occurs in condensed chromatin (56), (b) repair of (6–4)PPs is faster than repair of CPDs throughout the chromatin and most lesions are repaired within the first 4 h after UV, and (c) (6–4)PPs are preferentially repaired over CPDs in open chromatin during the rapid phase of NER (see Fig. 10). Furthermore, they showed that TC-NER of (6–4)PPs (see Regulation of DNA excision repair in transcriptionally active chromatin) is more efficient than for CPDs (56). Each of these observations agree with, and extend, the results of previous studies (discussed above).

Importantly, the impact of chromatin structural states on NER also correlates with mutation density in the genome of melanoma patients. “Closed” chromatin regions, which are repaired less efficiently by NER, are associated with high somatic mutations in melanomas (56). Moreover, a significant correlation exists between mutation density and chromatin accessibility in melanocytes (161). These results indicate that variable NER activity, which is dictated by open and closed chromatin states, plays an important role in determining global mutation heterogeneity in the melanoma genome.

Sancar and colleagues also determined the genome-wide kinetics of NER for (a) intrastrand crosslinks induced by cisplatin and (b) bulky DNA adducts induced by the carcinogen benzo[*a*]pyrene (27, 162). They found that, like the repair of CPDs, NER of both Pt-1,2-days(GpG) and BPDE-dG adducts is regulated by chromatin structure. High NER activity is associated with open chromatin states, such as gene promoters, enhancers, and transcribed genes, while low NER efficiency is observed in “closed chromatin” (27, 162), indicating that NER activity is modulated by the chromatin structural state, and *independent of DNA damage type*.

The Wyrick and Roberts groups at Washington State University used the CPD-seq method to examine NER efficiency of UV damage on a global chromatin level in yeast (58). They found that the translational setting of CPDs in NCPs plays a key role in the NER efficiency within nucleosomes. Specifically, CPDs located near the nucleosome dyad are repaired less efficiently than those located near the nucleosome ends. This “translational dependency” of NER is consistent with the fact that nucleosome dynamics are lowest in the dyad center region of NCPs and increase progressively toward the nucleosome DNA ends (43, 163).

The Wyrick and Roberts groups also used genome-wide maps of DNA base damage to follow repair and mutagenesis in MMS-treated yeast cells (164). They found that BER of the major MMS-induced alkylation product (7^me^G) is also significantly modulated by chromatin *in vivo*, with faster repair occurring in nucleosome-depleted regions, and slower repair *and* higher mutation density in strongly positioned nucleosomes. Analogous to the NER of CPDs, both the translational and the rotational settings of 7^me^G in NCPs significantly influence BER efficiency (164). It should also be noted that the minor alkylation product of MMS (3^me^A) is repaired so rapidly, it was unclear if nucleosomes affect their repair. Moreover, MMS-induced mutations at adenine nucleotides were significantly enriched on the NTS of yeast genes, particularly in BER-deficient strains, due to both higher damage formation on the NTS and the presence of TCR on the TS (164). These results revealed the influence of chromatin structure on BER and mutagenesis of base lesions in yeast and suggest a novel mechanism for “transcription-associated mutation asymmetry,” a frequently observed occurrence in human cancers [*e.g.*, (165)].

**Figure 12:**
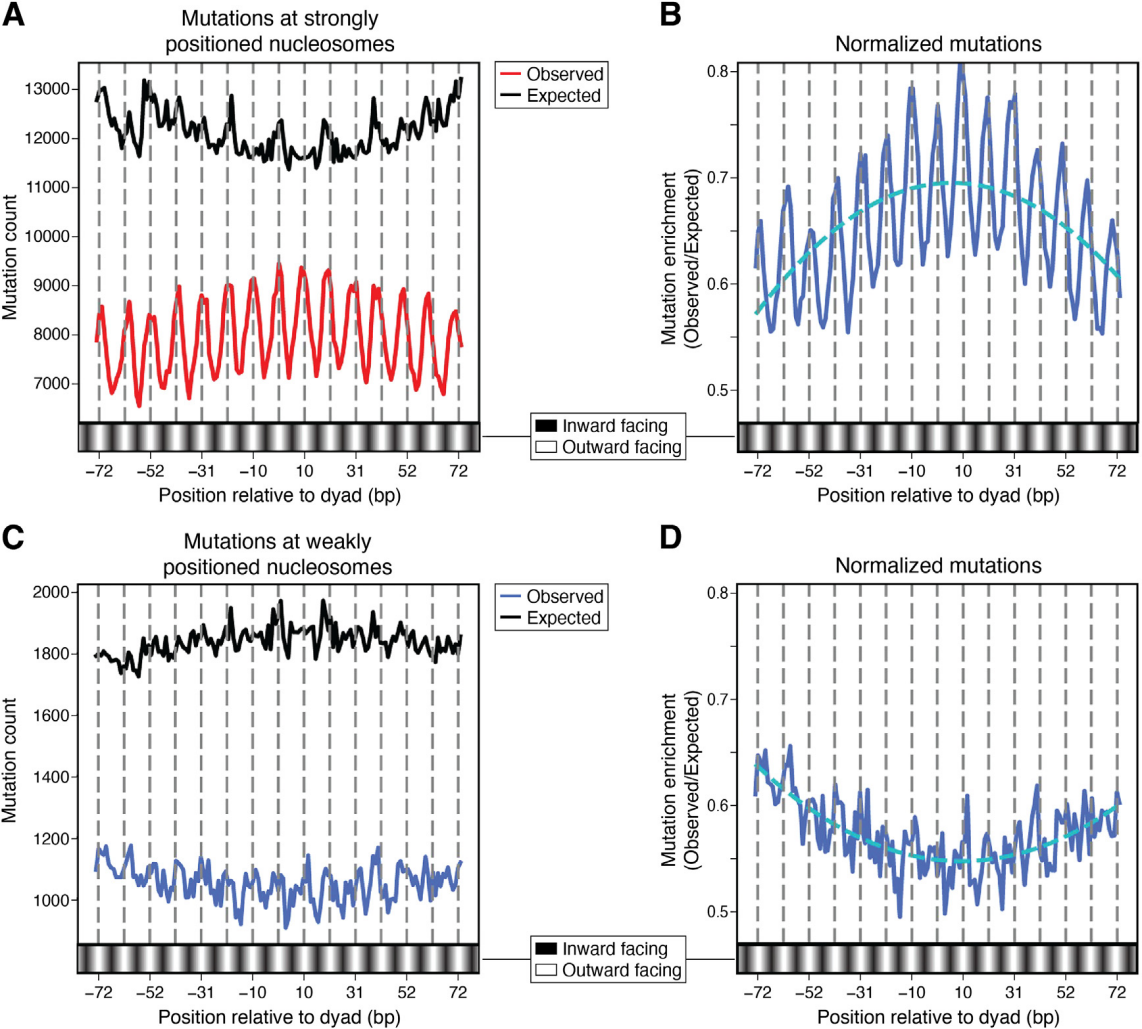
**Mutations in strongly positioned nucleosomes.***A*, observed single nt substitutions (*red line*) and expected mutations (*black line*) based on the sequence at individual bp across NCP positions. *Dashed lines* denote outward rotational settings of DNA, occurring every 10.3 bp. *B*, observed mutations normalized to expected mutations (*i.e.*, mutation enrichment or ME) display even more pronounced ∼10 bp periodicity and a “negative curvature” across the NCP. This curvature can be best fit by a second order polynomial (*dashed blue line*). *C*, observed (*blue line*) and expected (*black line*) mutations in weakly positioned nucleosomes. *D*, ME in weakly positioned nucleosomes (Modified from Figure 1 of (167)).

More recently, studies on the genome-wide role of nucleosome positioning and fine-structure in determining the *mutational distribution* in human cancers were reported (166, 167). The Wyrick and Roberts groups used CPD-seq, XR-seq, and high-sensitivity damage mapping data generated from NHFs (79, 160) to analyze the positions of melanoma mutations within strongly and weakly positioned nucleosomes (>1 million nucleosomes in each class) across the human genome. They found that, in strongly positioned nucleosomes, the mutation count and mutation enrichment (ME; observed/expected) in melanoma has a unique oscillatory pattern, with peaks occurring at 10.2 to 10.3 bp intervals at outward rotational settings in NCPs (Fig. 12*A*). Moreover, ME displays an enhanced ∼10 bp periodicity and has a negative curvature across the nucleosome (Fig. 12*B*). This curvature shows maximum ME values over the dyad region and falls off toward the NCP DNA ends.

Conversely, neither observed nor expected mutations showed an obvious pattern at weakly positioned nucleosome sites (Fig. 12*C*). The ME profile also didn’t show a significant pattern, and the curvature across the nucleosome was the opposite of strongly positioned nucleosomes (Fig. 12*D*). These results suggest that strongly positioned nucleosomes are associated with a unique mutation signature, having peaks in mutation density at outward rotational settings in NCP DNA with enrichment in mutations occurring near the NCP dyad axis.

The Wyrick and Roberts groups also analyzed the NER efficiency at different nucleosome positions after normalization to initial CPD levels (167). They found that NER is slower in DNA close to the dyad of strongly positioned nucleosomes (>1 million in humans) relative to the DNA at NCP ends. Thus, both the rotational and translational settings of DNA lesions in nucleosomes play an important role in modulating mutations in melanoma, albeit through different mechanisms. The pattern of CPD formation in NCPs likely plays a role in the ∼10 bp ME periodicity, while the variation in NER across strongly positioned NCPs likely plays a role in the “translational curvature” in the ME profile (Fig. 12*B*).

To test the origin of these mutational patterns, the Wyrick-Roberts groups repeated these analyses within strongly positioned nucleosomes of cutaneous (UV-exposed) and acral (typically not UV-exposed) melanoma subtypes (167). The ME profile in acral melanoma nucleosomes lacked the internal ∼10 bp oscillation and showed only a slight negative curvature across the NCP. In contrast, cutaneous melanoma mutations reflected the strong ∼10 bp oscillation and negative translational curvature in the ME profile, indicating that both are derived from UV damage.

Similarly, mutations occurring in dipyrimidine sequences of non-UV exposed prostate cancers also did not yield an oscillating ME profile. These results indicate that the oscillatory pattern of mutation density in nucleosomes is a unique feature of the UV-induced mutagenesis of cutaneous melanomas (167).

The Wyrick and Roberts groups also deconstructed nucleosomes by chromatin state, histone PTMs, and transcriptional status [see (167)] and found that the ME periodic profile persists in the NCPs of each of these subgroups. However, nucleosomes within different chromatin states or histone PTM states associated with active transcription displayed differences in the translational curvature of the NCP ME profile (167). These data indicate that the occupation time of nucleosomes on DNA may further dictate mutational density.

A “panoramic view” of the effects of nucleosomes on mutation rates, was reported by Lopez-Bigas and colleagues (166), who used high-resolution mapping of nucleosome positions in human cells (13) to map somatic mutations and germline variants in different human cell types. These authors found a striking periodic “mutation enrichment signal” repeating at ∼191 bp intervals (166), or close to the average nucleosome repeat length in human cells (9, 13). Interestingly, the *phase* of this periodic signal differs between tumor types, where high mutation rates are periodic in the NCPs of most tumors (*e.g.*, lung adenocarcinomas), mutation rates are enriched in linker regions in others (*e.g.*, skin melanomas) or have no clear periodic pattern (*e.g.*, ovarian cancer) (166).

**Figure 13:**
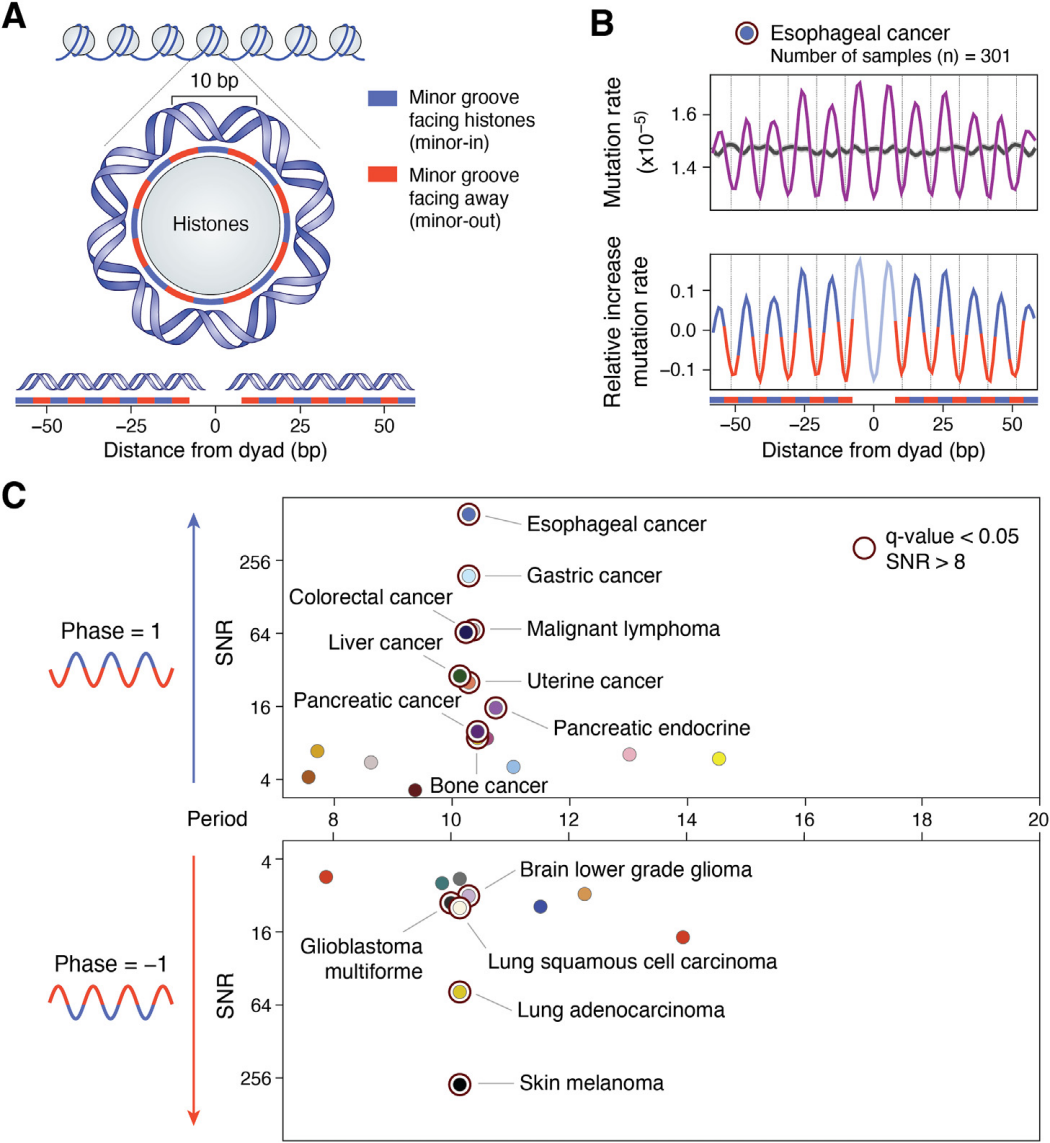
**Tumor Mutation Rate in Nucleosomes (Modified from**Figure 2**of** (166)**).***A*, schematic of DNA minor groove facing **In** and **Out** from histones. *B*, observed and expected mutation rate of esophageal adeno-carcinomas (*top*) and relative increase of mutation rate (RIMR, *bottom*). The periodicity of RIMR is 10.3 bases, and the phase shift of the signal at this period with respect to a reference sinusoidal signal with maxima at **In** facing minor grooves. For a RIMR with a phase shift of ∼ 0 radians, it was assigned a phase of 1, and a phase shift of ∼μ radians was assigned a phase of −1. Vertical *dashed lines* denote positions of minor groove facing **Out**. *C*, Signal-to-noise ratio (SNR, ordinate) of the strongest period (abscissa) in the RIMR of cohorts with phase 1 (*top*) or −1 (*bottom*). See (166) for details.

Analogous to the study by Brown *et al*. (167), Lopez-Bigas and colleagues performed analyses at high resolution within nucleosomes and found a strong ∼10 bp periodicity in somatic mutation rates in tumor cell NCPs (Fig. 13). The periodic pattern they observed also followed the oscillation of the DNA minor groove facing toward and away from the histones (Fig. 13, *A* and *B*), and the increase of mutation rate yielded a phase shift (relative to a reference sinusoidal signal) for most cancer types (Fig. 13*C*). Similar periodic patterns were seen in the genetic variation between humans and *Arabidopsis*, indicating the same principles hold for germline and somatic mutation rates (166). The authors hypothesized that DNA damage and repair processes are dependent on the minor groove orientation in NCP DNA and contribute to the ∼10-bp periodicity in AT/CG content in eukaryotic genomes.

The Lopez-Bigas group also deconstructed the contribution of distinct mutational signatures (as defined previously (165)) to each tumor and found that dominant signatures (associated with defined mutational processes) are major determinants of the observed phase periodicity in nucleosome-covered DNA. Combining mutations corresponding to each mutation signature revealed a strong correspondence between mutation signatures and the orientation of mutation-rate periodicity (166). Thus, these two seminal reports (166, 167), provide strong evidence that the interaction between different mutagenic agents and DNA repair mechanisms within nucleosomes govern unique mutation rate periodicities in human cells.

Another example of nucleosome fine structure modulating DNA repair and mutagenic profiles was recently reported by Wyrick’s group (119). Previously, it was assumed that inhibition of repair is equivalent on *both sides* of the nucleosome dyad (*i.e.*, whether going 5′ or 3′ from the DNA bp intersecting the dyad axis (see Fig. 2, left panel)). However, Wyrick’s group used genome-wide repair data to show that NER of UV damage in nucleosomes *is asymmetric*, by showing that faster repair of UV photoproducts occurs on the 5′ side of NCP DNA in the NTS of genes in both UV irradiated yeast and human cells (119). In contrast, the distribution of somatic mutations in nucleosomes revealed the opposite asymmetry in NER-proficient skin cancers, but not in NER-deficient cancers, suggesting that this asymmetric repair imposes a strand polarity on UV mutagenesis (119). Somatic mutations are enriched on the slower repairing 3′ sides of NCP DNA, especially at positions where the DNA minor groove faces away from the histone octamer. This asymmetric repair and mutagenesis are likely caused by differential accessibility to NCP DNA, a consequence of its left-handed wrapping around the histone octamer surface (119). Since somatic mutations occurring in melanoma driver genes are elevated in the slower repairing 3′ side of the NCP DNA (119), asymmetric repair in strongly positioned nucleosomes may have important implications for carcinogenesis.

## Alteration in chromatin structure during DNA excision repair

**Figure 14:**
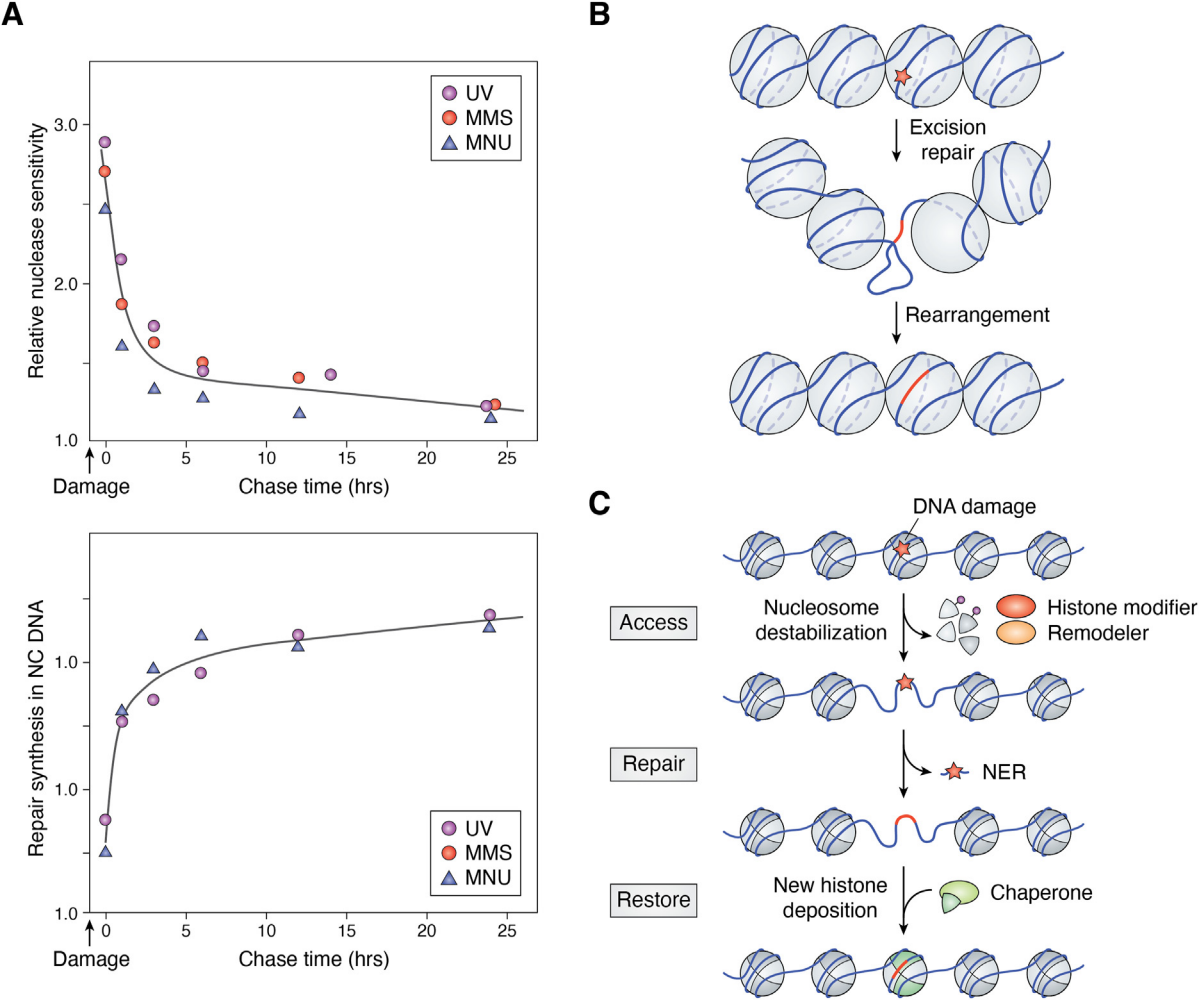
**Evolution of a model.***A*, nuclease sensitivity of newly repaired DNA in chromatin of NHFs (*upper panel*) and repair synthesis in isolated NCPs (*lower panel*) following treatment with UVC, MMS and methylnitrosourea (MNU). Cells were pulse-labeled with [^3^H]dThd after damage treatment and chased for times shown (Modified from Figure 3 of (6)). *B*, original “unfolding–refolding” model for NER in chromatin (170). *C*, access-Repair-Restore (ARR) model of nucleosome rearrangements during NER of damaged chromatin in mammalian cells (242). Symbols: DNA, *blue line*, repair synthesis, *red line*, histone modifications, *purple solid circles*, core histones, *light & dark grey*, and newly synthesized histones, *green* (Modified from Fig. 1*C* of (238)).

Evidence for the rearrangement of chromatin structure following DNA damage emerged almost 50 years ago from studies by Lieberman’s lab (117). These observations were inspired by earlier work from both the Cleaver and Lieberman groups, who had examined the accessibility of newly repaired DNA, labeled with [^3^H]dThd, to MNase in chromatin of UV-irradiated NHF cells (112, 114). These initial studies revealed that regions that had just undergone repair synthesis were more rapidly digested by MNase than the bulk of the DNA in chromatin (112, 114). One conclusion from these findings was that NER synthesis occurred preferentially in nuclease-accessible regions of chromatin (*e.g.*, nucleosome linker DNA) and remained nuclease sensitive, leading to the idea of a non-uniform distribution of NER in chromatin (112). An alternative explanation, however, came from the surprising result that the nuclease accessibility of newly repaired regions *quickly changed* over time in the cell (Fig. 14*A*) (117). “Nucleosome rearrangement” in newly repaired regions was revealed by both the loss of nuclease sensitivity of newly repaired DNA and the reassociation of newly repaired DNA with canonical nucleosome structures during increasing chase times (Fig. 14*A*). Since the time course of these changes was very similar, it was apparent that these two phenomena were associated with different aspects of the same structural changes occurring at the nucleosome level in chromatin. Similar results were obtained with DNase I digestions (42, 114, 168), including restoration of the canonical ∼10 base “ladder” on denaturing gels (9).

During the ensuing decade, different laboratories observed nucleosome rearrangement following repair synthesis induced by a variety of different DNA damaging agents, from bulky chemicals that form adducts preferentially in linker DNA to methylating agents which have nearly equal access to the linker and core DNA [see (169)]. As nucleosome rearrangement follows a biphasic time course (Fig. 14*A*), including an early rapid phase (representing nucleosome reassembly) and a late slow phase (involving nucleosome repositioning) (42), these findings led to the original “unfolding–refolding” model (Fig. 14*B*) reported by Lieberman and coworkers in 1979 (42, 170). This model underwent several refinements over the years as new data was obtained [*e.g.*, (6, 171)] and depicts rearrangement as the rapid refolding of newly repaired DNA into a canonical nucleosome structure after an initial unfolding of this region for processing by DNA repair enzymes (Fig. 14*C*).

Recent studies using high-resolution fluorescent imaging of chromatin components in intact cells indicate there are rapid changes in both the structural constraints and the nucleosome occupancy following UV-induced DNA damage (172, 173). These changes are stimulated by the binding of DDB2 at UV-damaged sites and result in increased mobility of large domains of the damaged chromatin (174). Furthermore, fluorescence microscopy studies of UV-irradiated hamster cells revealed that DDB2 elicits this chromatin decompaction in an ATP-dependent manner, which coincides with a poly(ADP ribose) polymerase-dependent reduction in core histone density near the lesion (175). Additionally, Polo and colleagues at the Université de Paris used real-time tracking of parental H3 and H4 histones after localized UV damage in human cells to identify a conservative process where parental histones rapidly redistribute away from UV-damaged chromatin and subsequently recover (172). The restoration of chromatin structure at the damage sites ensued via chromatin re-compaction and sliding of nucleosomes bearing the parental histones. This process was tightly coupled to the progression of NER through the binding and release of DDB2 (172). A model where parental histones remain in the vicinity of UV-damaged sites to allow restoration of chromatin structure after NER was proposed (172).

Histone chaperones also play a key role in the DDR. For example, Polo’s group analyzed the dynamics of histone variants in the chromatin of UV-damaged human cells and discovered there is a turnover of histone variants H2A.Z and H2A.X that is controlled by the histone chaperones ANP32 E (acidic nuclear phosphoprotein 32 family member E) and FACT (176). They found that newly synthesized H2A.X is deposited by FACT at UV-damage sites in a NER-dependent manner and this activity is preceded by H2A.Z removal by ANP32E. Furthermore, the deposition of H2A.X at repair sites was independent of H2A.X phosphorylation (forming γH2AX), a key activity for amplifying DNA damage signaling (177). As H2A.Z increases chromatin compaction *in vitro* (178) and forms a complex with HP1*a* (heterochromatin protein 1, isoform *a*) that directs assembly of structurally distinct heterochromatin (179), depletion of H2A.Z from UV-damaged chromatin may contribute to the early relaxation of chromatin discussed earlier. Given these results, Polo and coworkers proposed that ANP32E removes H2A.Z from chromatin-damaged sites to enhance the accessibility of these regions to DNA repair proteins and, subsequently, FACT promotes new H2A.X deposition coupled to NER synthesis (176). This change in the chromatin landscape could promote DNA damage signaling and contribute to the cascade of repair proteins at damage sites in chromatin.

## Regulation of DNA excision repair in transcriptionally active chromatin

### Excision repair of RNA Pol II genes

**Figure 15:**
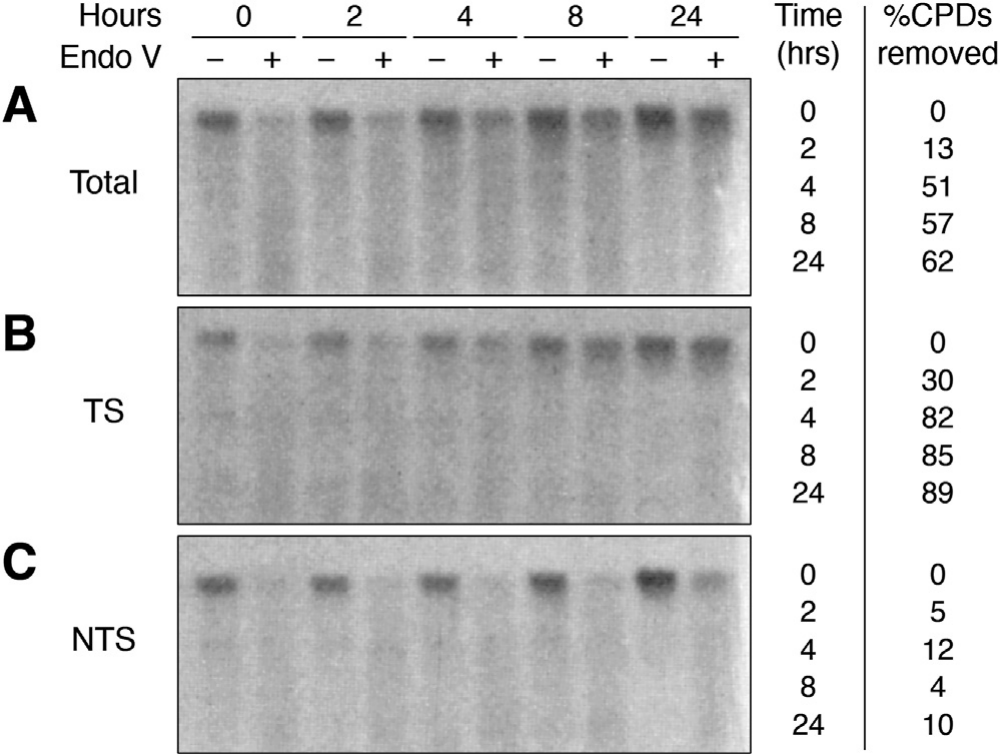
**Preferential Repair of the *Dhfr* Gene in CHO cells (Modified from**Figure 2**and**Table 1**of** (183)**).** Genomic DNA was isolated at times indicated after UV irradiation and digested with *Kpn*l. Samples were treated with (+) or without (−) T4 endo V to cleave the DNA specifically at CPDs and electrophoresed on denaturing gels. The *Kpn*l fragment was detected with a ^32^P-labeled DNA probe to detect both DNA strands (*A*) or a ^32^P-labeled RNA probe to detect either the TS (*B*) or the NTS (*C*). See (183) for details.

Preferential repair of transcriptionally active genes in chromatin was first reported by Hanawalt and coworkers at Stanford University (180). These investigators initially found that repair of CPDs in UV-irradiated mammalian cells is more efficient in the active dihydrofolate reductase (*Dhfr*) gene than in neighboring transcriptionally silent regions of chromatin (181, 182). They then used strand-specific probes to demonstrate that preferential repair of CPDs occurs on the TS of the *Dhfr* gene in both CHO and human cells (Fig. 15) (183). These ground-breaking studies were rapidly followed by reports showing that TC-NER is also present in UV-irradiated *E. coli* (184) and yeast (185), and recently reported in UV-irradiated halophilic *Archaea* (186) and *Arabidopsis* (187). Furthermore, TC-NER of different bulky DNA lesions (CPD, cisplatin, and psoralen) has been demonstrated in a completely defined system *in vitro* both biochemically and at the single molecule level using purified bacterial proteins (22). Thus, TC-NER appears to stand as a universal DDR for the repair of bulky DNA lesions in the TS of transcriptionally active chromatin, spanning across different eukaryotic species and biological kingdoms.

The TC-NER pathway is initiated by the stalling of elongating RNA Pol II at bulky, helix-distorting DNA lesions (188). The first responders, CSA-CSB complex and UV-sensitive syndrome protein (UVSSA), contribute to the processing of blocked RNA Pol II and the recruitment of NER factors in mammalian cells (29). These activities initiate the unwinding and excision of the lesion-containing ssDNA fragment, which is followed by repair synthesis and ligation (189). A central player in this process was shown to be RNA Pol II transcription factor TFIIH (190, 191). As reviewed by Egly and colleagues, recruitment of TFIIH is critical in this process and several of the TFIIH subunits have now been shown to have direct roles in NER (21).

Several non-lesion barriers, such as altered DNA structures, also block Pol II elongation (192), and this raises the question of how cells distinguish between different forms of arrested Pol II to commit TC-NER only to those blocked by DNA lesions. Mechanistic insight was provided by Wang and colleagues who solved the cryo-EM structure of the *S. cerevisiae* RNA Pol II-Rad26 elongation complex (193). These investigators found that Rad26 promotes the forward motion of Pol II in an ATP-dependent manner (193). However, when the translocation blockage is strong RAD26 cannot promote efficient transcriptional bypass. Thus, these data suggest a model where only the interaction between Rad26 and Pol II that is strongly blocked at a DNA lesion would lead to the initiation of TC-NER (193).

Transcriptionally active regions of chromatin have unique structural features that allow increased accessibility to the DNA (14). Thus, it was contemplated early on that these features may play a role in the preferential repair of active chromatin (171). Smerdon and Thoma exploited the use of yeast minichromosomes to study the repair of transcriptionally active chromatin in intact cells (8, 32).

These plasmids could be designed to allow accurate mapping of repair rates at specific sites in nucleosomes and transcriptionally active genes (121, 185, 194). This system also benefited from the extensive genetics establishing numerous NER genes in yeast (4, 195).

The minichromosome TRURAP contains a single selectable gene (*URA3*), an autonomous origin of replication (*ARS1*), and nucleosomes of known position and stability (196). Also, the overall rate of NER in UV-treated TRURAP is similar to that of genomic chromatin (121). This is the case for the repair of CPDs in *wt*, *rad*1, and *rad*7 yeast cells (197); although this correlation was mysteriously absent in *rad*23 cells (198). Smerdon and Thoma measured repair at over 40 different CPD sites in TRURAP and found that repair rates vary markedly along the plasmid (185). Rates were highest in the TS of *URA3* and in both strands upstream of this gene, while being lowest in the NTS of *URA3* and both strands of the ARSl region. Next, it was found that four different (presumably) nonsense transcripts are also made from TRURAP, *in addition* to *URA3* mRNA (194). These transcripts encompass *all* the efficiently repaired regions outside the *URA3* gene, and there was a good correlation between the rates of transcription and rates of repair in four of the five transcribed regions (194). The fifth region, which is very weakly transcribed in yeast cells, is rapidly repaired and contains two nucleosomes that are much less stable (194, 199). This latter result was the first example of (a) a lack of correlation between repair and transcription rates and (b) the regulation of NER by nucleosome stability in a transcribed region.

Following these reports, Waters and Reed at Cardiff University carried out a series of systematic studies on the mechanisms of repair of UV photoproducts in yeast chromatin (200). These investigators initially examined NER at individual CPDs in the *MFA2* gene of *S. cerevisiae*, which produces the mating-type factor a2 (201). This gene is silent with a heterochromatin structure in ***a*** mating-type cells but is active with an open chromatin structure in **a** mating-type cells (202). Surprisingly, in addition to the TS bias for NER in transcribing *MFA2*, enhanced repair was also observed in the control region, upstream of the transcription start site in active *MFA2* (201). This region was found to be only partially repaired in *RAD16* mutants (201), implicating the Rad7/Rad16 complex in the repair of the *MFA2* gene promoter. Subsequently, it was shown that the Rad7/Rad16 complex does indeed participate in the repair of non-transcribed regions (4, 203).

The Cardiff group went on to isolate the Rad7/Rad16-containing GG-NER complex and found it to have DNA translocase activity; although, unlike many SWI/SNF superfamily complexes, this complex was not able to slide nucleosomes along DNA *in vitro* (204). The Rad7 and Rad16 proteins form a stoichiometric complex (205) that binds damaged DNA in an ATP- dependent manner (206). In addition, Rad7 is part of an E3 ligase complex that ubiquitinates Rad4, a core NER protein in yeast, that binds damaged DNA (207). Importantly, ubiquitination of Rad4 was shown to directly influence NER and UV survival (207, 208).

Another protein that co-purified with Rad7 and the GG-NER complex was transcription factor Abf1 (207). In the absence of UV damage, Abf1 forms a stable heterotrimeric complex with Rad7 and Rad16 and about a third of the total cellular Abf1 was predicted to be associated with this complex (209). Originally, Abf1 was identified for both its ability to bind DNA replication origins and its role in silencing the HML and HMR *loci* of *S. cerevisiae* (210). Subsequently, Abf1 was shown to bind upstream activating sequences (UASs) of a variety of different gene promoters, and it is now well established that this protein is an essential, global site-specific DNA binding protein (207, 211). These results led to a proposed mechanism for NER and chromatin rearrangement at the *MFA2* locus, which includes Abf1 in the initiation complex (209). This model accounts for enhanced NER of the UAS and maintenance of a repressed state following repair [see (200, 207)].

### Excision repair of RNA Pol I and Pol III genes

Measurement of DNA repair in the multi-copied ribosomal or tRNA genes of eukaryotes is complex because only a fraction of these genes is transcriptionally active at one time (212). Furthermore, this fraction can change with cell type (from ∼20% to ∼80%) and, at least in the yeast *S. cerevisiae*, with growth conditions (213). In addition, there are structural differences between RNA Pol I and RNA Pol II stalled at a CPD in the TS that could play a role in defining a possible coupling between transcription and repair in these genes (214).

Initial reports found there was inefficient repair of both psoralen interstrand cross-links and UV-induced CPDs in the total rDNA of mammalian cells (215, 216). Furthermore, there was no evidence for strand-specific repair in total rDNA (216, 217). It was also reported that there is no repair of CPDs in the ribosomal genes of human XPC cells (*i.e.*, cells lacking GG-NER) and lower than normal repair of CPDs in the rDNA of CSA and CSB cells (*i.e.*, lacking TC-NER) (218). This latter result implies that either the NER deficiency in rDNA of CS cells is not due to a defective TC-NER factor (218) or a subset of active ribosomal genes are repaired by TC-NER.

**Figure 16:**
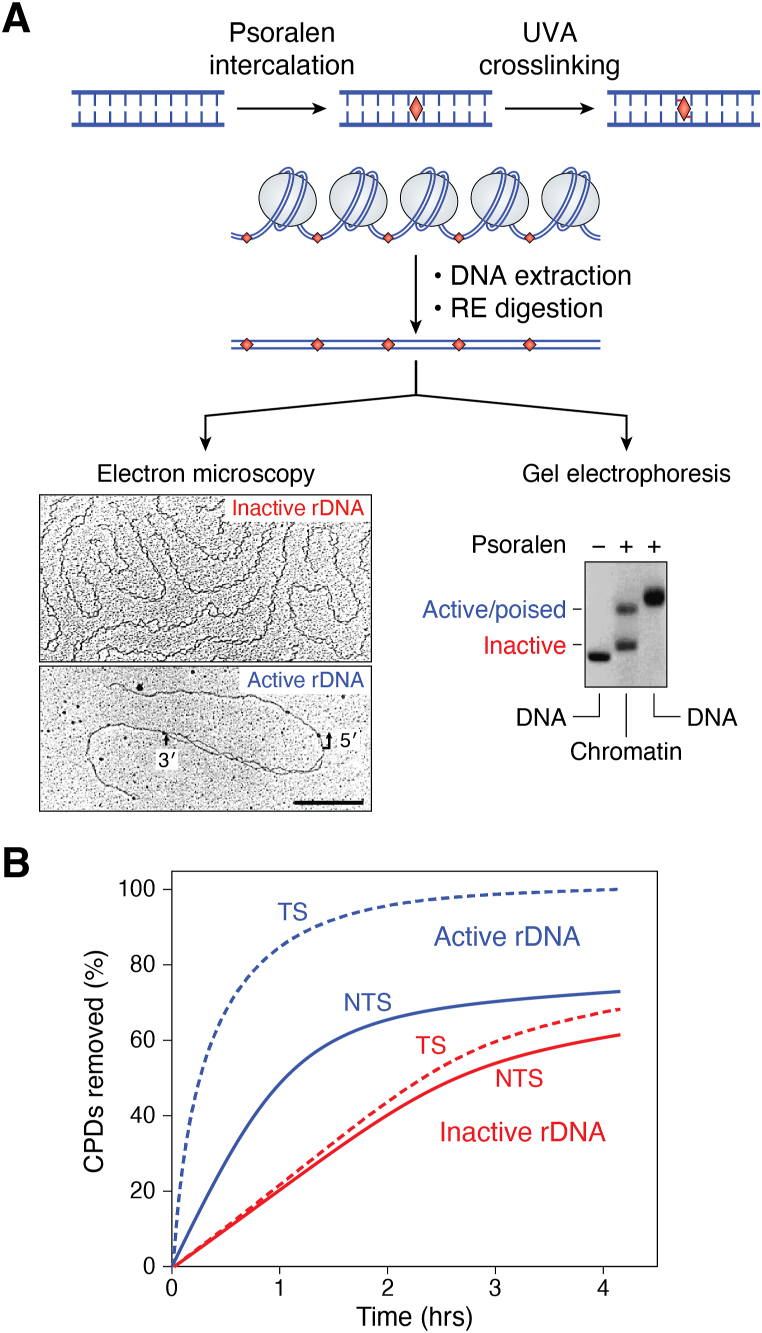
**Transcription-coupled repair of the ribosomal genes in yeast.***A*, separation of the transcriptionally active and inactive fractions of ribosomal genes by psoralen crosslinking. The TMP crosslinking scheme and EM data are from Dr José Sogo, Swiss Federal Institute of Technology (ETH) (see (39) for details), and gel electrophoresis data are from (221). *B*, repair of the individual strands of active and inactive rDNA chromatin from UV-irradiated *S. cerevisiae* (Modified from Figure 7 of (229)).

Sogo and colleagues at the Swiss Federal Institute of Technology developed biochemical methods to complement their EM studies that separated the transcriptionally active and inactive rDNA fractions based on their differing sensitivity to psoralen crosslinking and restriction enzyme digestion [for review, see (219, 220)] (Fig. 16*A*). Smerdon’s group used these methods to allow the direct measurement of CPD removal from each strand of the active ribosomal genes in mouse erythroleukemia cells (221), the same cells used previously by Sogo’s group, to thoroughly characterize ribosomal gene chromatin by EM and psoralen crosslinking (222). However, even after isolation of the active rDNA fraction, TC-NER was not observed in these genes, and repair of UV-induced CPDs was diminished in each strand (221). These results supported the previous notion that TC-NER does not exist in mammalian rDNA.

Recently, the question of transcription-repair coupling in mammalian ribosomal genes was revisited with advanced genomics technologies. One study, using SV40-immortalized human fibroblasts, reported that TC-NER repairs UV-induced lesions in the rDNA of these cells and this activity is dependent on the CSA, CSB, and UVSSA genes while being independent of the XPC gene and that rDNA repair takes place at the periphery of the nucleolus in these cells (223). On the other hand, Sancar and coworkers used 45S pre-rRNA sequences and novel bioinformatic programs for sequence alignments to map NER in the rDNA of human and mouse cell lines (224). Using data generated by the XR-seq method, no evidence for preferential repair of CPDs in the TS of rDNA in telomerase-immortalized human fibroblasts was found. Nonetheless, the results indicated that UV-induced DNA lesions were repaired in human rDNA. Namely, repair of the TS and NTS is comparable in both WT and CSB mutant cell lines, while it is abolished in each strand in an XPC mutant cell line (224). It is important to note, as Pol I transcription is “hyperactive” in cancer cells (225, 226), transcription of rDNA in the SV40-immortalized human fibroblasts (223) may also be hyperactive. The stress response in these cells may then require TC-NER to handle the damage load and support cell survival. Therefore, both the extent of repair of CPD lesions and the participation of TC-NER in the nucleolus of higher eukaryotes remains unclear. It is possible that these inconsistencies reflect differences in ribosomal gene transcription frequency, which is cell-line and cell growth dependent.

In contrast to mammalian cells, yeast cells have been shown to efficiently remove UV- induced CPDs from their rDNA *via* the NER pathway (Fig. 16*B*). Moreover, early studies by Brouwer and colleagues at Leiden University observed modest, yet significant, strand-specific repair in the total rDNA of *S. cerevisiae* cells (227). More pronounced preferential repair of the TS in Rad7 and Rad16 mutants, which contribute to the repair of non-transcribed DNA, was observed. These data were the first report that TC-NER may exist in RNA Pol I transcribed genes in a eukaryotic organism. Notably, this preferential repair of the TS of rDNA was independent of Rad26, an important factor in most TC-NER events in RNA Pol II transcribed genes (227, 228).

More recently, the Conconi and Thoma groups showed that repair of rDNA in yeast displays a strand bias in the actively transcribing rDNA fraction of chromatin, but not in the inactive fraction (212, 229, 230) (Fig. 16*B*). These results confirmed and extended the results of Brouwer’s group (227) by demonstrating that efficient NER of the TS of rDNA occurs in the actively transcribing fraction of ribosomal chromatin, satisfying the operational definition of TC-NER. Surprisingly, it was found that strand-specific repair of rDNA is not eliminated in *rad4* cells (227) and TC-NER is totally operational in the active rDNA fraction (231). As RAD4 mutants are defective in the incision step of NER and Rad4 is essential for both GG-NER and TC-NER in yeast (232, 233), this was unexpected. Still, this result may reflect another observation by the Brouwer group that the Rad34 protein, which shares homology with Rad4, is essential for preferential repair in the TS of rDNA but has no apparent role in the repair of Pol II transcribed genes (234). For example, the Rad4 protein is needed for the removal of UV photoproducts in the intergenic spacer region of rRNA genes, as well as, in both strands of inactive rDNA and the NTS of active rDNA (231). Moreover, TC-NER starts 40 nts downstream of the TSS and Rad4 is needed for the removal of photoproducts in the TS *before* this position (231). On the other hand, Rad34 is needed for TC-NER downstream of the TSS (231). Thus, although Rad4 and Rad34 share sequence homology, their roles are different but complementary and it remains to be seen if TC-NER of Pol I transcribed genes is a unique feature of yeast or is present in the rDNA of mammalian cells. Given these results, Conconi’s group proposed a model for the fate of Pol I and nucleosomes at UV-damaged sites, which predicts that TC-NER and GG-NER could combine in a spatiotemporal relationship for handling the repair of active rDNA genes in yeast (235). Recently, the Conconi group reported that the two NER sub-pathways ‘inversely participate’ in the removal CPDs from the TS, where in the NTS of both nucleosome and non-nucleosome rRNA gene coding regions, GG-NER is solely responsible for removing UV-induced DNA lesions (236).

## Conclusion and perspectives

In summary, this journey began in 1972 when, after obtaining a graduate degree in physics, MJS joined Dr Irvin Isenberg’s lab at Oregon State University to study the physical properties of histone H1 subfractions. Rumblings of a major finding in molecular biology (*i.e.*, the discovery of the fundamental unit of chromatin) had already begun as there were several landmark papers in the early to mid 1970s that laid the groundwork for the nucleosome model published in 1974 by Roger Kornberg (237) (For extensive accounts of this period, see (9, 10).). These results set the stage for the initial experiments on DNA damage and repair in chromatin. The preference of different DNA damaging agents to react with nucleosome linker regions (7, 22) was not surprising but the marked bias of damage for the DNA strand facing away from the histone surface (43, 47), as well as the modulation of damage yield in TFBS (79, 80), was unexpected. These results led to a much better understanding of the mutation profiles in human cancers, some of which may be drivers of neoplastic transformation (50, 79, 80, 119, 167). In addition, the regulation of DNA excision repair in chromatin held more surprises. The disruption of nucleosomal DNA (47), and higher order chromatin organization during repair (172, 173, 174), especially for NER, indicated there is chromatin remodeling during DNA repair in chromatin (93, 207, 238). Indeed, to the best of our knowledge, the original observation of this process in 1978 (117) was the first example of chromatin remodeling occurring in cells. This activity also limited the assessment of the distribution of excision repair in chromatin because it rendered all actively repairing regions much more accessible to nuclease digestion, the most common method for determining chromatin distribution at that time (171). Then came the advance of genome-wide mapping of DNA damage and repair during the last decade. These methods gave us a global view of the distribution of damage and repair at the nucleotide level (50, 51) and have revolutionized our understanding of DNA damage, DNA repair, and mutagenesis (50, 56, 58, 119, 22, 187). Furthermore, these studies have led to a better understanding of the connection(s) between mutagenesis and human disease (including cancer).

Thus, in 50 years we evolved from a blurred view of how DNA is packaged into cell nuclei to how this packaging regulates the formation of DNA damage, the repair of this damage, and the fate of chromatin regulation on mutagenic profiles in human cells. Although it is hard to imagine how far this field will advance in the next half-century, one of the themes of this period will certainly be the epigenetic responses serving as triggers in chromatin during the DDR, as well as, the reconstitution of *complete* segments of chromatin fibers (*e.g.*, polynucleosomes) containing specific histone H1 subfractions and nonhistone proteins (*e.g.*, high mobility group proteins HMGA, HMGB and HMGN (239)) for well-controlled *in vitro* studies. However, perhaps the most significant findings during the next half century will be complete surprises.

## Dedication

We dedicate this review to Irvin Isenberg and Kensal van Holde, Professors of Biochemistry & Biophysics at Oregon State University, whose pioneering research played a major role in the discovery of the nucleosome and fostered our initial studies on DNA repair in chromatin. MJS is forever grateful for their guidance and exceptional mentorship during his graduate training.

## Conflict of interest

The authors declare that they have no known competing financial interests or personal relationships that could have appeared to influence the work reported in this paper.
